# Long-Distance Axon Regeneration Promotes Recovery of Diaphragmatic Respiratory Function after Spinal Cord Injury

**DOI:** 10.1523/ENEURO.0096-19.2019

**Published:** 2019-09-26

**Authors:** Mark W. Urban, Biswarup Ghosh, Cole G. Block, Laura R. Strojny, Brittany A. Charsar, Miguel Goulão, Sreeya S. Komaravolu, George M. Smith, Megan C. Wright, Shuxin Li, Angelo C. Lepore

**Affiliations:** 1 Department of Neuroscience, Vickie and Jack Farber Institute for Neuroscience, Sidney Kimmel Medical College at Thomas Jefferson University, Philadelphia, PA 19107; 2 Department of Neuroscience, Shriners Hospitals for Pediatric Research Center, Temple University School of Medicine, Philadelphia, PA 19140; 3 Department of Biology, Arcadia University, Glenside, PA 19038

**Keywords:** cervical, diaphragm, phrenic, plasticity, PTEN, sprouting

## Abstract

Compromise in inspiratory breathing following cervical spinal cord injury (SCI) is caused by damage to descending bulbospinal axons originating in the rostral ventral respiratory group (rVRG) and consequent denervation and silencing of phrenic motor neurons (PhMNs) that directly control diaphragm activation. In a rat model of high-cervical hemisection SCI, we performed systemic administration of an antagonist peptide directed against phosphatase and tensin homolog (PTEN), a central inhibitor of neuron-intrinsic axon growth potential. PTEN antagonist peptide (PAP4) robustly restored diaphragm function, as determined with electromyography (EMG) recordings in living SCI animals. PAP4 promoted substantial, long-distance regeneration of injured rVRG axons through the lesion and back toward PhMNs located throughout the C3–C5 spinal cord. These regrowing rVRG axons also formed putative excitatory synaptic connections with PhMNs, demonstrating reconnection of rVRG-PhMN-diaphragm circuitry. Lastly, re-lesion through the hemisection site completely ablated functional recovery induced by PAP4. Collectively, our findings demonstrate that axon regeneration in response to systemic PAP4 administration promoted recovery of diaphragmatic respiratory function after cervical SCI.

## Significance Statement

Using non-invasive and only transient systemic delivery of an antagonist peptide that targets phosphatase and tensin homolog (PTEN), we promoted significant regeneration of bulbospinal respiratory axons into and through a large lesion site in a rat model of cervical spinal cord injury (SCI), as well as growth of these axons back into the intact distal spinal cord for several spinal segments. We also found that these regenerating axons synaptically reconnected with their appropriate postsynaptic respiratory motor neuron targets, resulting in reconnection of the damaged neural circuit. Furthermore, by showing that re-lesion of regenerating axons resulted in complete loss of PTEN peptide-induced diaphragm recovery, we demonstrate that long-distance axon regeneration can drive recovery of respiratory function following cervical SCI.

## Introduction

**Figure 1.**
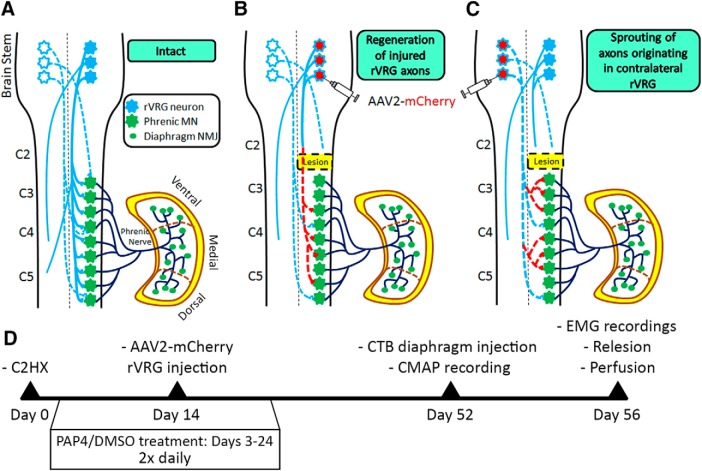
Schematic of the rVRG-PhMN-diaphragm circuit. Schematic of intact (***A***), ipsilateral regeneration of ablated rVRG fibers (***B***), and contralateral rVRG fiber sprouting (***C***). Blue lines represent descending rVRG axons and red dotted lines represent regrowing axons. ***D***, Experimental timeline: C2 hemisection was performed on all animals, and from days 3 to 24 after injury, animals were treated with twice-daily subcutaneous injections of either DMSO or PAP4. Two weeks following injury, animals received either ipsilateral or contralateral brainstem injections of AAV2-mCherry. Four days before killing, CMAP recordings were taken for each animal and CTB injections into the ipsilateral hemidiaphragm were performed. At eight weeks after injury, EMG recordings were taken, and then animals were killed to collect brain and spinal cord tissue samples.

Traumatic spinal cord injury (SCI) produces many debilitating outcomes, including respiratory compromise (Lane et al., 2008a). A majority of human SCI cases occur in the cervical spinal cord, resulting in persistent diaphragm dysfunction that is associated with mortality, a host of morbidities such as respiratory infections, and greatly reduced quality of life (Warren and Alilain, 2014; Warren et al., 2014). Diaphragm is directly controlled by phrenic motor neurons (PhMNs) located at cervical levels C3–C5 (Lane et al., 2009). PhMNs are mono-synaptically activated by supraspinal brainstem neurons located in the rostral ventral respiratory group (rVRG; Fig. 1*A*; Dobbins and Feldman, 1994). Cervical SCI results in axotomy of descending rVRG fibers, denervation and consequent silencing of spared ipsilateral PhMNs, and partial-to-complete hemidiaphragm paralysis (Fig. 1*B*; Urban et al., 2018). Restoration of rVRG-PhMN-diaphragm circuitry therefore represents a critically important therapeutic target for individuals affected by SCI.

Promoting the combination of robust regeneration of damaged rVRG axons and synaptic reconnection of these growing axons with their PhMN targets is a major goal for addressing diaphragmatic respiratory compromise following cervical SCI. However, various inhibitory mechanisms limit/prevent robust axonal growth and ultimately the synaptic reconnectivity that will mediate functional restoration after CNS injury. These include (1) neuronal-intrinsic factors that limit the ability of both injured and spared CNS neurons to mount an axon growth response (Luo and Park, 2012) and (2) environmental impediments to axon growth such as an acellular cystic cavity, the molecular barrier presented by the glial scar, myelin-associated inhibitory molecules, and repulsive developmental axon guidance molecules that continue to be expressed in the adult CNS (Bradbury et al., 2002; Giger et al., 2010).

Blocking environmental sources of regenerative inhibition in SCI models results in only limited axonal regrowth, suggesting the importance of modulating intrinsic growth capacity of neurons (Huebner and Strittmatter, 2009). The phosphatase and tensin homolog (PTEN)-mechanistic target of rapamycin (mTOR) axis is a key pathway that is involved in the developmental down-regulation of axon growth capacity (Park et al., 2010). Experimentally modulating this signaling system has been shown to successfully promote axon regrowth in SCI animal models, including plasticity of patient-relevant neuronal populations such as corticospinal tract axons (Park et al., 2010; Luo and Park, 2012; Liu et al., 2015). The axon growth response to molecules such as neurotrophic factors occurs in part via receptor-mediated activation of Akt. This results in downstream activation of mTOR, thereby initiating a pro-growth state by regulating mechanisms such as, for example, gene transcription and protein translation in the neuronal soma and cytoskeletal dynamics in axons (although additional processes likely are also involved; Park et al., 2010; Kanno et al., 2012). PTEN is a phosphatase that prevents Akt activation by converting PIP_3_ to PIP_2_. Inducible genetic knockout of PTEN (Park et al., 2008; Liu et al., 2010; Willenberg et al., 2016) or viral vector-based shRNA knockdown (Zukor et al., 2013; Lewandowski and Steward, 2014) increases CNS axonal growth after injury, while mTOR inhibition blocks the pro-growth effects of genetic PTEN deletion, demonstrating that PTEN puts a brake on the neuron’s ability to signal through mTOR.

In the current work, we tested systemic administration of a PTEN antagonist peptide (PAP) in a rat model of high-cervical (level C2) SCI (Urban et al., 2018), a novel approach to promote plasticity of rVRG neurons and restoration of rVRG-PhMN-diaphragm circuitry. We previously developed a number of PAPs directed against various regions of the PTEN protein (Ohtake et al., 2014). These peptides bind directly to and inhibit the activity of PTEN. The PAP used in the current study is a 30 amino acid peptide (including the TAT sequence for facilitating entry into cells) that targets the C-terminal tail of PTEN. We chose to use this particular PAP (termed PAP4) because it results in the most robust axon growth in the thoracic dorsal funiculotomy SCI model. Specifically, we have previously shown that systemic PAP4 delivery promotes plasticity of injured corticospinal tract axons, enhances serotonergic fiber growth and results in partial recovery of hindlimb motor function (Ohtake et al., 2014). Importantly, PAP4 cross the blood-brain barrier and enters CNS parenchyma after systemic injection.

PAP4 is a powerful tool both experimentally and therapeutically for manipulating axon growth capacity after SCI. PAP4 has therapeutic potential given that it can be delivered in a non-invasive, systemic manner and for defined temporal duration (Ohtake et al., 2014). PAP4 also allows us to examine the effects of modulating PTEN function, and more generally neuronal-intrinsic growth inhibition, on rVRG axon growth and respiratory recovery after SCI. Lastly, PAP4 provides an *in vivo* experimental tool to study the contribution of defined modes of axonal growth and rVRG-PMN circuit reconnectivity to recovery of function following SCI (i.e., regeneration of injured axons originating in the ipsilateral rVRG vs sprouting of spared fibers originating in the contralateral rVRG).

In this study, we show that systemic administration of PAP4 to cervical SCI rats promotes robust, long-distance regeneration of injured bulbospinal rVRG axons and synaptic reconnection of these regrowing fibers with their PhMN targets, which was associated with significant recovery of diaphragmatic respiratory function.

## Materials and Methods

### Animal model

Female Sprague Dawley rats weighing 250–300 g were purchased from Taconic Farms. All rats were housed three animals per cage in temperature, humidity and light controlled environments with ad libitum access to food and water. Experimental procedures were approved by the Thomas Jefferson University IACUC and conducted in compliance with Animal Research: Reporting of In Vivo Experiments (ARRIVE) guidelines and the Society for Neuroscience’s Policies on the Use of Animals and Humans in Neuroscience Research.

### Postoperative care

Following survival surgeries, muscle layers were sutured with 4–0 silk sutures (Covidien) and the skin was closed with surgical staples (Braintree Scientific). The surface of the skin was treated with a topical iodine solution. Immediately following the procedure, rats were treated with 5-ml subcutaneous injections of lactated Ringer’s solution (Hospira), buprenorphine hydrochloride (0.05 mg/kg; Hospira) and cefazolin (6 mg; Hospira). Rats were then placed in a clean cage on a surgical heating pad set to 37°C (Gaymar). At 12 and 24 h after surgery, each rat was given an additional dose of buprenorphine hydrochloride (0.05 mg/kg) and 5 ml of lactated Ringer’s solution and monitored for pain/distress.

### C2 hemisection SCI

Rats were anesthetized with an intraperitoneal injection of ketamine HCl (95.0 mg/kg; Vedco), xylazine (10.0 mg/kg; Lloyd Laboratories), and acepromazine (0.075 mg/kg; Phoenix Pharm Inc). Using a #11 surgical blade (Electron Microscopy Sciences), a one-inch midline incision was made on the dorsal surface of skin and muscle to expose the dorsal surface of the C2 and C3 vertebrae. A laminectomy was performed above C2 to expose the spinal cord using rongeurs (Fine Science Tools). A hemisection was performed just caudal to the C2 root with a dissecting knife (Fine Science Tools; Urban et al., 2018). To ensure a complete hemisection, a 30-gauge needle (BD Biosciences) was passed through the injury several times.

### rVRG injection

Rats were anesthetized with a mixture of ketamine/xylazine/acepromazine, as described above. Using a #11 surgical blade, a half-inch incision was made on the dorsal surface of the skin at the occipital bone and superficial muscles were separated with surgical scissors (Fine Science Tools) and a retractor (Fine Science Tools), exposing the occipital bone and C1 vertebrae. Rongeurs were used to remove the ligament between the occipital bone and the C1 vertebrae, followed by removal of the caudal portion of the occipital bone to reveal the obex. The animals were then placed on a stereotaxic frame (Kopf Instruments). Using the obex as a starting point, the rVRG was located by the following coordinates: 2.0 mm lateral, 1.0 mm rostral, and 2.6 mm ventral. An UltraMicroPump (World Precision Instruments) injection system was used to inject 0.3 μl of total volume of AAV2-mCherry with a microsyringe (Hamilton) attached to a Micro4 Microsyringe Pump Controller (World Precision Instruments; Urban et al., 2018). Immediately following injection, the needle was left in place for 5 min before being slowly removed from the medulla.

### Diaphragm injection

Rats were anesthetized with a mixture of ketamine/xylazine/acepromazine. A laparotomy was performed starting from the xyphoid process and cutting laterally just below the rib cage to expose the right hemidiaphragm. Once exposed, cholera toxin subunit B recombinant, Alexa Fluor 647 conjugate (Life Technologies) was injected into the ventral, medial and dorsal portions of the hemidiaphragm (5 μl injected per site), specifically targeting the endplate band (Li et al., 2015b).

### Electromyography (EMG) recordings

Once the animals were deeply anesthetized with ketamine/xylazine/acepromazine, a laparotomy was performed to expose hemidiaphragm. Bipolar electrodes spaced 3 mm apart were placed for recording in three separate subregions of the hemidiaphragm: dorsal, medial and ventral. Recordings during normal eupnic breathing were averaged over a continuous 60-s time frame for each animal, and peak amplitude, burst duration and frequency were taken (Nicaise et al., 2012, 2013). Every inspiratory burst over these 60 continuous seconds was included in the analysis. Using LabChart 7 software [ADInstruments; Research Resource Identification Initiative RRID: SCR_001620], the EMG signal was amplified and filtered through a bandpass filter (50–3000 Hz).

### Compound muscle action potential (CMAP) recordings

Rats were anesthetized with isoflurane (Piramal Healthcare) at a concentration of 3.0–3.5% diluted in oxygen. Animals were placed supine, and the region just below the rib cage was shaved and cleaned with 70% ethanol. Phrenic nerve conduction studies were performed with stimulation of the phrenic nerve via needle electrodes transcutaneously inserted into the neck of the rat in proximity to the passage of the phrenic nerve (Lepore et al., 2008, 2010). A reference electrode was placed on the shaved surface of the right costal region. The phrenic nerve was stimulated with a single burst at 6 mV (amplitude) for a 0.5-ms duration. Each animal was stimulated between 10 and 20 times to ensure reproducibility, and recordings were averaged for analysis. ADI Powerlab8/30 stimulator and BioAMP amplifier (ADInstruments) were used for both stimulation and recording, and Scope 3.5.6 software (ADInstruments; RRID: SCR_001620) was used for subsequent data analysis. Following recordings, animals were immediately euthanized with a dose of Euthasol, and tissue was collected (as described below).

### Spinal cord and brainstem dissection

Animals were euthanized with Euthasol. After the right hemidiaphragm was excised, the animal was exsanguinated by cutting the right atrium. Next, the animal was transcardially perfused with 0.9% saline solution (Fisher Scientific), followed by 4% paraformaldehyde (Electron Microscopy Sciences) to fix the tissue. Following perfusion, the spinal cord and brain were excised with rongeurs (Fine Science Tools) and kept in a 4% paraformaldehyde solution overnight at 4°C, washed with 0.1 M phosphate buffer [sodium phosphate dibasic heptahydrate (Sigma-Aldrich) and sodium monobasic monohydrate (Sigma-Aldrich)], and placed in 30% sucrose (Sigma-Aldrich). Samples were then submerged in an embedding mold (Polysciences, Inc) and covered with tissue freezing medium (General Data). Samples were then flash frozen in 2-methylbutane (Fisher Scientific) chilled in dry ice. Tissue was sectioned on a cryostat (Thermo Scientific). Brainstem (transverse) and spinal cord (sagittal) sections were cut at 30 μm, placed on glass slides (Fisher Scientific), dried overnight, and stored at –20°C for histologic analysis.

### Immunohistochemistry

Before immunostaining, tissue sections were dried for 1 h at room temperature. Antigen retrieval was performed using R&D Systems Protocol (R&D Systems). Immediately after antigen retrieval, a hydrophobic pen was used to surround the tissue sections (Newcomer Supply). Slides were blocked/permeabilized for 1 h at room temperature with a solution of 5% normal horse serum (Vector Laboratories), 0.2% Triton X-100 (Amresco), diluted in PBS (primary and secondary antibodies were diluted in this solution as well). Slides were then treated with primary antibody overnight at 4°C with the following antibodies: neuronal marker anti-NeuN at 1:200 (EMD-Millipore; RRID: AB_2298772), astrocyte marker anti-GFAP at 1:400 (Dako; RRID: AB_10013482), oligodendrocyte lineage marker anti-Olig-2 at 1:200 (EMD-Millipore; RRID: AB_2299035), anti-synaptophysin antibody at 1:250 (Abcam, Cambridge, MA; RRID:AB_2198854), anti-5HT antibody at 1:500 (Immunostar; RRID:AB_572263), anti-phospho-S6 ribosomal protein (Ser235/236) at 1:50 (Cell Signaling Technology; RRID:AB_331679), anti-wheat germ agglutinin (WGA) at 1:50 (Vector Laboratories; RRID:AB_2315608), anti-VGLUT2 at 1:250 (EMD-Millipore; RRID:AB_2187552), anti-DsRed at 1:500 (Clontech Laboratories, Inc.; RRID:AB_10013483), anti-CTB at 1:10,000 (List Biological Laboratories). On the following morning, samples were washed 3× in PBS, and secondary antibody solutions were added for 1 h at room temperature: donkey anti-rabbit IgG H&L (Alexa Fluor 647) at 1:200 (Abcam), donkey anti-mouse IgG H&L (Alexa Fluor 488) at 1:200 (Abcam), rhodamine (TRITC) AffiniPure donkey anti-goat IgG (H + L) at 1:200 (Jackson ImmunoResearch). Following secondary antibody treatment, samples were washed in PBS, and two to three drops of FluorSave reagent (Calbiochem) were added to tissue sections, then slides were coverslipped (Fisher Scientific). Slides were stored at 4°C.

### Spinal cord histology

Tissue sections were dried at room temperature for 2 h. Slides were then placed in 3-min baths of xylene, 100% ethanol, 95% ethanol, 70% ethanol, and dH_2_O. Slides were next placed in an eriochrome solution (0.16% eriochrome cyanine, 0.4% sulfuric acid, 0.4% ferric chloride in dH_2_O) for 14 min, washed with tap water, placed in a developing solution (0.3% ammonium hydroxide in dH_2_O) for 5 min, washed with dH_2_O, and then placed into a cresyl violet solution (0.4% cresyl violet, 6% 1 M sodium acetate, and 34% 1 M acetic acid) for 18 min. Finally, slides were placed in baths of dH_2_O, 70% ethanol, 95% ethanol, 100% ethanol, and xylene. Slides were then mounted with poly-mount xylene (Polysciences), and cover slips were added. Slides were then kept at room temperature for analysis.

### pS6K70 immunofluorescence quantification

Brainstem sections were immunostained with anti-phospho-S6 ribosomal protein (Ser235/236), followed by donkey anti-mouse IgG H&L (Alexa Fluor 488) at 1:200 (Abcam). Individual mCherry+ rVRG neurons were traced manually in ImageJ/Fiji software (RRID:SCR_003070), using images captured via 20x objective on a Zeiss Axio M2 Imager (Carl Zeiss Inc.; Urban et al., 2018). Mean intensity per mCherry+ cell was measured using ImageJ/Fiji software. Average background intensity values were subtracted from individual neurons to obtain each intensity value. Average neuronal intensities were recorded and resulting values were quantified using three tissue sections per animal, and three animals were used per group. For pS6K quantification, measurements of individual neurons were not averaged within an animal to produce a single value for a given animal. We choose to perform the analysis in this manner as we observed significant neuron-to-neuron variability in pS6K levels within an animal. Obtaining a single mean value for the entire animal does not reflect the shift we saw in individual neurons across the entire population within an animal. Importantly, changes in this intracellular signaling can have significant effects on functional outcome even if only a subset of these rVRG neurons have alterations in PTEN signaling and consequent effects on axonal growth capacity.

### rVRG axon regeneration quantification

To detect mCherry anterograde tracer expression, 30-μm sagittal sections of spinal cord tissue were imaged with a Zeiss Axio M2 Imager (Carl Zeiss Inc.). The high-magnification images were then optically “stitched” using MetaMorph software (Molecular Devices; RRID:SCR_002368) to generate a complete image of the cervical spinal cord. The stitched images were rostral-caudally binned in 100-μm segments using the rostral border of the injury and rostral intact spinal cord as the starting point (Urban et al., 2018). All mCherry-labeled axons were counted; axons that traversed through more than one bin were counted in all bins that they traversed.

### Analysis of putative synaptic contacts between rVRG axons and PhMNs

To assess putative excitatory synaptic connections between presynaptic mCherry+/VGLUT2+ rVRG axon terminals and postsynaptic CTB+ PhMNs, we immunostained sagittal sections with antibodies against DsRed, CTB, and VGLUT2 (Urban et al., 2018). For quantification of connections at level C3 ipsilateral to lesion, we considered contacts in which the mCherry+/VGLUT2+ terminal was located within 2.5 μm of the surface of the CTB+ PhMN cell body in single-z confocal images, based on previous work (Kinkead et al., 1998; Issa et al., 2010). PhMN membrane surface area was not considered in this analysis.

### Quantification of rVRG axon sprouting

Here, 30-μm sagittal tissue sections from cervical spinal cords of animals injected with AAV2-mCherry into the contralateral rVRG were immunostained with anti-DsRed antibody (1:200). mCherry-labeled axon profiles were counted in the ventral horn ipsilateral to the injury at C3, C4, and C5 regions using a standard area size of analysis for every section, at each spinal cord level and in all animals. Five sections of tissue were analyzed per animal and 4 animals per group were quantified. Results are expressed mean ± SEM.

### 5-HT analysis

For quantitative analysis of 5-HT immunostaining, we used the same image acquisition parameters for all samples, including excitation light intensity, camera settings and exposure time. In sagittal sections, we quantified number of 5-HT axon profiles, total 5-HT axonal length and integrated intensity of 5-HT immunolabeling within the CTB-labeled PhMN pool ipsilateral to the injury at C3–C5 (Ghosh et al., 2018). To assess the number of putative synaptic connections between presynaptic 5-HT+/synaptophysin+ serotonergic axon terminals and postsynaptic CTB+ PhMNs, we immunostained sagittal sections with antibodies against 5-HT, CTB and synaptophysin. For quantification at levels C3, C4 and C5 ipsilateral to the lesion, we considered contacts in which the 5-HT+/synaptophysin+ terminal was located within 2.5 μm of the surface of the CTB+ PhMN in single-z confocal images (Ghosh et al., 2018).

### Diaphragm dissection

Animals were euthanized with Euthasol (Virbac AH Inc.) and then placed in a supine position. Two incisions were made into the skin and underlying muscle to expose the right hemidiaphragm. The right hemidiaphragm was excised using spring scissors (Fine Science Tools), stretched flat and pinned down on silicon-coated 10-cm dishes, and washed with PBS (Gibco). Diaphragms were then fixed for 20 min in 4% paraformaldehyde (Electron Microscopy Sciences). After washing in PBS, superficial fascia was carefully removed from the surface of the diaphragm with Dumont #5 Forceps (Fine Science Tools). Diaphragms were then stained for neuromuscular junction (NMJ) markers (see NMJ analysis).

### Diaphragm whole-mount histology

Fresh hemidiaphragm muscle was dissected from each animal for whole-mount immunohistochemistry, as described previously (Wright and Son, 2007; Wright et al., 2007, 2014). Diaphragms were rinsed in PBS and then incubated in 0.1 M glycine for 30 min. Following glycine incubation, α-bungarotoxin conjugated to Alexa Fluor 555 at 1:200 (Life Technologies) was used to label postsynaptic nicotinic acetylcholine receptors. Ice-cold methanol was then added to the diaphragms for 5 min, and then diaphragms were blocked for 1 h at room temperature in a solution of 2% bovine serum albumin and 0.2% Triton X-100 diluted in PBS (this solution was used for both primary and secondary antibody dilutions). Primary antibodies were added overnight at 4°C: presynaptic vesicle marker anti-SV2 at 1:10 (Developmental Studies Hybridoma Bank; RRID: AB_2315387), neurofilament marker anti-SMI-312 at 1:1000 (Covance; RRID:AB_2314906). The diaphragms were then washed and secondary antibody solution was added for 1 h at room temperature: FITC anti-mouse IgG secondary (Jackson ImmunoResearch; 1:100). Diaphragms were mounted with Vectashield mounting medium (Vector Laboratories), coverslips were added, and slides were stored at –20°C.

### NMJ analysis

Labeled muscles were analyzed for the percentage of NMJs that were intact, partially denervated, or completely denervated (Wright and Son, 2007; Wright et al., 2007, 2014). Whole-mounted diaphragms were imaged on a FluoView FV1000 confocal microscope (Olympus; RRID:SCR_014215). We conducted NMJ analysis on ipsilateral hemidiaphragm as our previous work indicated no denervation or sprouting in the contralateral hemidiaphragm after cervical SCI (Nicaise et al., 2012).

### Glial scar quantification

Here, 30-μm sagittal tissue sections of cervical spinal cord were immunostained with anti-GFAP antibody (as described above). The rostral-to-caudal width of the GFAP+ scar was measured on a Zeiss Axio M2 Imager using MetaMorph software at five equidistant locations starting at the dorsal end of the hemisection and going to the ventral end of the injury to account for changes in scar size through the dorsal-ventral extent of the tissue. At each location, scar width measurements were calculated starting at the edge of the lesion (i.e., the location where the GFAP+ scar meets the GFAP– lesion site) and extending through the dense, continuous band of interdigitated astrocytes with highly-upregulated levels of GFAP expression. These glial scar measurements were collected on both rostral and caudal sides of the lesion. All 10 measurements per single section (5 on each side of the lesion) were then averaged, and each animal had 10 tissue sections analyzed to calculate a single mean value for glial scar width per animal (*n* = 3 animals per group).

### Lesion quantification

Here, 30-μm sagittal tissue sections of cervical spinal cord were stained with cresyl violet (as described above). The lesion was measured on a Zeiss Axio M2 Imager using MetaMorph software at five equidistant locations starting at the dorsal end of the hemisection and going to the ventral end of the injury to account for changes in lesion size through the tissue (Urban et al., 2018). All five measurements per section were averaged, and each animal had 12 sections of tissue analyzed to determine a value for lesion size per animal (*n* = 3 animals per group).

### Experimental design and statistical analysis

In the Results section, we provide details of exact *n*s, group means, SEM, statistical tests used, and the results of all statistical analyses (including exact *p* values, *t* values, and *F* values) for each experiment and for all statistical comparisons. Statistical significance was assessed by ANOVA and multiple comparisons *post hoc* test; *t* test was used for analysis involving only two groups. GraphPad Prism 6 (GraphPad Software Inc.; RRID: SCR_002798) was used to calculate all analyses, and *p* < 0.05 was considered significant. We replicated this study in two completely separate experiments conducted approximately nine months apart. Importantly, we observed the same outcome with both the functional EMG experiments and the analysis of rVRG axon regeneration in the two studies, demonstrating reproducibility of the robust effects of PAP4 administration after cervical SCI.

We authenticated relevant experimental regents to ensure that they performed similarly across experiments and to validate the resulting data. We generated the AAV2-mCherry vector in-house. Whenever we used a new batch of the vector, we verified that the virus performed equivalently from batch-to-batch by confirming in every animal that the vector transduced cells of the rVRG, transduced predominantly NeuN-positive neurons and induced expression of the mCherry tracer in the soma and axonal projections of these cells. If viral delivery was unsuccessful because of mis-targeting of the intra-medullary injection or because lack of mCherry expression, we would not use this animal for analysis of the rVRG axonal growth response. We histologically verified lesion completeness of the C2 hemisection in all animals. If a rat showed an incomplete lesion, the animal would be completely excluded from the study for all analyses. Whenever a new batch of peptide was used (i.e., in the two replication experiments), we validated that systemic administration of PAP4 was able to increase levels of pS6K70 in the soma of rVRG neurons using immunostaining of brainstem sections. For Alexa-conjugated α-bungarotoxin and for all antibodies used in the immunohistochemistry studies, we always verified (when receiving a new batch from the manufacturer) that labeling in the spinal cord, brainstem and/or diaphragm muscle coincided with the established expression pattern of the protein. We have provided RRID numbers for all relevant reagents (i.e., antibodies and computer programs) throughout Materials and Methods.

## Results

### C2 hemisection disrupted rVRG-PhMN-diaphragm circuitry

Under intact physiologic conditions, inspiratory signals originating from the rVRG are mono-synaptically relayed to PhMNs of the C3–C5 spinal cord that directly innervate the ipsilateral hemidiaphragm (Fig. 1*A*; Dobbins and Feldman, 1994). There is also some bulbospinal input to PhMNs from the contralateral rVRG. After C2 hemisection, rVRG fibers are axotomized, resulting in hemidiaphragm paralysis (Urban et al., 2018). To test the effect of PTEN inhibition on rVRG axon regeneration, we subjected adult female Sprague Dawley rats to C2 hemisection SCI (Alilain et al., 2011), followed by 2× daily subcutaneous injections of PAP4 or DMSO-only control starting 3 d after injury and continuing for 21 d (Fig. 1*D*). At two weeks after injury, we performed intra-medullary injection of an AAV2-mCherry vector to anterogradely-label rVRG axons (Urban et al., 2018). In separate cohorts, we transduced neurons of either the ipsilateral rVRG to assess regeneration of damaged axons (Fig. 1*B*) or the contralateral rVRG to test for sprouting of spared fibers within the PhMN pool ipsilateral to the lesion (Fig. 1*C*). Four days before killing, we performed CMAP recordings from both the ipsilateral and contralateral hemidiaphragm to examine functional innervation of the muscle by PhMNs (Lepore et al., 2008, 2010). We also performed intrapleural injection of the retrograde tracer, cholera toxin subunit B, at this same time point to selectively label PhMN soma within the cervical spinal cord (Li et al., 2014, 2015a). At eight weeks following SCI, we conducted EMG recordings from anesthetized rats under normal eupnic breathing conditions to assay functional diaphragm activation and rhythmic bulbospinal drive to the PhMNs (Nicaise et al., 2012, 2013). A subset of PAP4-treated and DMSO-treated animals underwent a re-lesion at eight weeks after injury to determine whether axon regeneration through the injury site was causally responsible for functional diaphragm recovery; in this experiment, EMG recordings were performed on the same animal at eight weeks after injury both before and after the re-lesion procedure. All animals were killed at eight weeks after SCI.

### Increased mTOR signaling in rVRG neurons

**Figure 2.**
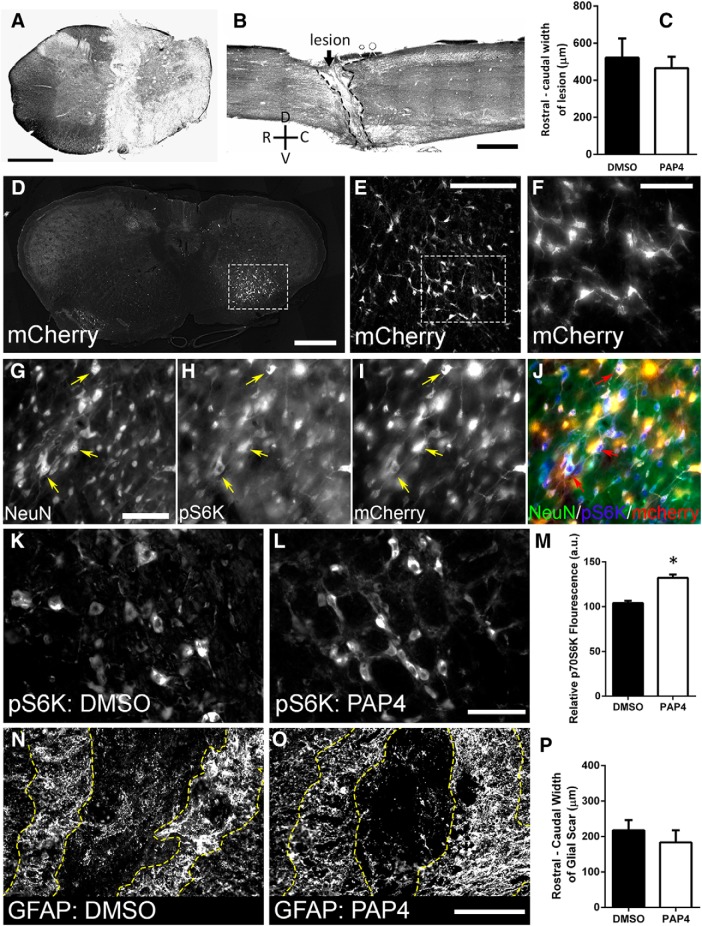
Increased mTOR signaling in rVRG neurons. At eight weeks after hemisection, cresyl violet staining of both transverse (***A***) and sagittal (***B***) sections shows complete dorsal-ventral and medial-lateral ablation of the spinal cord. No difference was observed between DMSO and PAP4 groups in rostral-to-caudal lesion size (***C***); scale bars: 1 mm (***A***), 1 mm (***B***). Fluorescent microscopy of coronal brainstem sections shows AAV2-mCherry injections into the rVRG (***D***); higher magnification images reveal robust expression of mCherry and neuronal morphology of transduced cells in rVRG (***E***, ***F***); scale bars: 1 mm (***D***), 500 μm (***E***), and 100 μm (***F***). mCherry+ coronal brainstem sections immunostained with NeuN and pS6K show co-localization of pS6K in NeuN+ neurons within the rVRG (***G–J***); scale bar: 100 μm. At eight weeks after injury, immunofluorescence imaging shows that PAP4-treated animals had significantly higher levels of pS6K in ipsilateral rVRG neurons compared to DMSO-treated controls (***K***, ***L***); scale bar: 100 μm; total fluorescence per mCherry+ cell was averaged for each animal and the mean of each group is represented in ***M***. Immunofluorescence imaging revealed no change in GFAP+ glial scar size at eight weeks after hemisection between DMSO (***N***) and PAP4 (***O***) groups; scale bar: 200 μm. Yellow dotted lines represent the rostral and caudal borders of the glial scar at the lesion site. Quantification of glial scar width shown in ***P***; *n* = 10 sagittal sections per animal and *n* = 3 animals per group for quantification. *p < 0.05.

We first verified complete hemisection in all animals by performing cresyl violet staining on both sagittal and transverse sections of injured spinal cord. We found complete medial-to-lateral (Fig. 2*A*) and dorsal-to-ventral (Fig. 2*B*) ablation of the hemicord, indicating complete disruption of descending rVRG axons within the ipsilateral spinal cord. We found no difference in the average width of the lesion site as determined by the distance from the rostral to the caudal lesion borders between the DMSO controls and the PAP4 group (DMSO: 521 ± 51.9 μm, *n* = 4; PAP4: 466 ± 30.1 μm, *n* = 4; *p* = 0.39, *t* test; Fig. 2*C*).

We next evaluated the efficiency of transduction of rVRG neurons using the AAV2-mCherry tracer. Transverse tissue sections of the brainstem revealed robust expression of mCherry within the rVRG (Fig. 2*D*), with viral transduction restricted to cells displaying a neuronal morphology (Fig. 2*E*,*F*). Using this same AAV2-mCherry vector, we previously showed that ∼95% of transduced cells within the rVRG are NeuN-expressing neurons (Urban et al., 2018).

We next tested the effect of PAP4 on the mTOR signaling axis. mTOR pathway stimulation results in downstream phosphorylation and activation of S6 kinase (S6K; Kanno et al., 2012). We previously showed that pS6K levels in ipsilateral rVRG neurons are significantly decreased following C2 hemisection compared to laminectomy-only (Urban et al., 2018). We first determined pS6K expression within mCherry-labeled neurons of the rVRG by immunostaining mCherry+ brainstem sections with NeuN and pS6K (Fig. 2*G–J*). We found that pS6K immunostaining co-labeled with NeuN-positive rVRG neurons that also were transduced by the AAV2-mCherry vector. We quantified pS6K fluorescence intensity per mCherry+ rVRG neuron specifically and found a significant increase in pS6K levels in the PAP4 group (Fig. 2*K*) compared to DMSO control (Fig. 2*L*) at eight weeks after injury [DMSO: 104 ± 2 (a.u.), *n* = 129 rVRG neurons; PAP4: 132 ± 4 a.u., *n* = 118; *p* < 0.0001; *t* test; Fig. 2*M*), indicating that systemic PAP4 delivery was effective in activating the mTOR pathway in rVRG neurons. These data likely underestimate the effect given that we stopped PAP4 delivery at three weeks after injury, while we quantified pS6K levels at eight weeks after hemisection. While we observed increased pS6K levels in rVRG neurons with PAP4, we have not shown that this effect of PAP4 is directly on rVRG neurons, particularly considering the long duration between the end of peptide treatment and the time of analysis.

### Glial scar formation was not affected by PAP4

The glial scar plays a critical role after SCI in both limiting secondary damage (Faulkner et al., 2004) and inhibiting axon growth (Silver et al., 2015). As glial scar formation after a severe insult such as hemisection SCI involves significant cell proliferation (Faulkner et al., 2004) and because PTEN plays a central role in the control of cell division, we assessed whether systemic PAP4 treatment influenced scar formation or lesion size, effects that could indirectly influence the rVRG axon growth response. To evaluate astrocyte scar formation, we immunostained sagittal spinal cord sections with the astrocyte marker GFAP. On both rostral and caudal sides of the lesion, we quantified the rostral-to-caudal width of the GFAP-positive scar at five equidistant locations (five locations on both sides) along the dorsal-ventral expanse of the injury. Compared to DMSO-only control (Fig. 2*N*), PAP4 (Fig. 2*O*) did not alter the width of the astroglial scar (DMSO: 217 ± 28.9 μm, *n* = 3; PAP4: 183 ± 34.1 μm, *n* = 3; *p* = 0.49; *t* test; Fig. 2*P*). These data are suggestive of a lack of PAP4 effects on scar formation; nevertheless, additional experiments are necessary to more conclusively rule out the possibility that PAP4 impacted the glial scar response.

### Diaphragm function was significantly restored by PAP4

**Figure 3.**
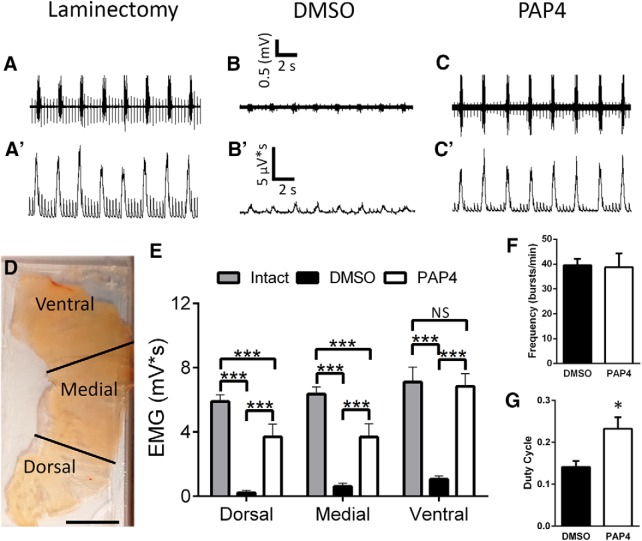
Diaphragm function was significantly restored by PAP4 following C2 hemisection. At eight weeks after injury, EMG recordings were assessed from uninjured, DMSO-treated hemisection, and PAP4-treated hemisection groups. EMG amplitudes were recorded at three subregions of the hemidiaphragm: dorsal, medial, and ventral regions (***D***). Both DMSO-treated and PAP4-treated groups were compared to intact (laminectomy-only) animals. Representative EMG traces from the ventral region of the diaphragm show that compared to laminectomy-only animals (***A***, ***A’***), DMSO-treated rats (***B***, ***B’***) had a significant decrease in inspiratory EMG burst amplitude at eight weeks after injury. Animals treated with PAP4 (***C***, ***C’***) showed significant recovery of EMG amplitude following injury. Quantification of EMG recordings at the three hemidiaphragm regions (***E***). Ventral subregion EMG burst frequency was unaffected by PAP4 treatment (***F***). EMG duty cycle was increased by PAP4 (***G***). *p < 0.05. **p < 0.01. ***p < 0.001. ns, not significant *p* ≥ 0.05.

To test for diaphragmatic functional recovery, we performed EMG recordings in anesthetized living animals from three different subregions of the diaphragm: dorsal, medial, and ventral (Fig. 3*D*). We and others have previously shown that the ventral subregion is primarily innervated by PhMNs located at C3, the medial subregion is controlled by C4 PhMNs, and the dorsal subregion is innervated by PhMNs residing at C5 (Laskowski and Sanes, 1987; Li et al., 2014). Compared to DMSO-only control (Fig. 3*B*,*B’*), PAP4 promoted significant functional recovery (Fig. 3*C*,*C’*) at all three subregions of the ipsilateral hemidiaphragm (Fig. 3*E*). In the dorsal (intact: 5.9 ± 0.4 mV/s, *n* = 9; DMSO: 0.2 ± 0.14 mV/s, *n* = 8; PAP4: 3.7 ± 0.8 mV/s, *n* = 9) and medial (intact: 6.4 ± 0.4 mV/s, *n* = 9; DMSO: 0.6 ± 0.2 mV/s, *n* = 8; PAP4: 3.7 ± 0.8 mV/s, *n* = 9) subregions, PAP4-induced recovery was partial as the EMG amplitudes were less than uninjured laminectomy-only rats (dorsal: *F*_(2,23)_ = 27.15; Tukey’s *post hoc* test; intact vs DMSO: *p* = 0.0002; intact vs PAP4: *p* = 0.2; DMSO vs PAP4: *p* = 0.03; medial: *F*_(2,23)_ = 24.5; Tukey’s *post hoc* test medial: intact vs DMSO: *p* < 0.001; intact vs PAP4: *p* = 0.10; DMSO vs PAP4: *p* = 0.06; ANOVA; Fig. 3*A*,*A’*,*E*), while PAP4 promoted complete recovery back to uninjured control levels in the ventral subregion (ventral: intact: 7.1 ± 0.9 mV/s, *n* = 9; DMSO: 1.6 ± 0.2 mV/s, *n* = 8; PAP4: 6.8 ± 0.8 mV/s, *n* = 8; *F*_(2,22)_ = 20.9; Tukey’s *post hoc* test; intact vs DMSO: *p* < 0.0001; intact vs PAP4: *p* = 0.97; DMSO vs PAP4: *p* < 0.0001; ANOVA; Fig. 3*E*). Additionally, PAP4 had no effect on EMG burst frequency (DMSO: 40 ± 2.6 bursts/min, *n* = 4; PAP4: 39 ± 5.5 bursts/min, *n* = 4; *p* = 0.90, *t* test; Fig. 3*F*), while EMG duty cycle was significantly increased with treatment (DMSO: 0.14 ± 0.01, *n* = 4; PAP4: 0.23 ± 0.01, *n* = 4; *p* = 0.004, *t* test; Fig. 3*G*) in the ventral subregion. These findings demonstrate that systemic delivery of PAP4 (starting with a delay of 3 d after injury) promoted significant recovery of diaphragmatic respiratory function after cervical SCI.

### Functional and morphologic innervation of the diaphragm was unaffected by PAP4

**Figure 4.**
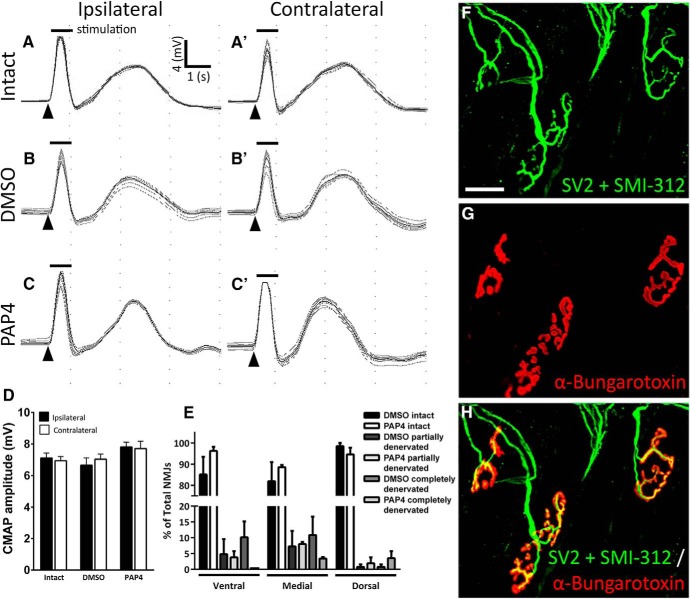
Functional and morphologic innervation of the diaphragm was unaffected by PAP4. CMAP recordings obtained from the ipsilateral hemidiaphragm show no differences in amplitude among laminectomy-only (***A***), DMSO (***B***), and PAP4 (***C***) groups. Similarly, no differences were noted in CMAP amplitudes obtained in the contralateral hemidiaphragm among laminectomy-only (***A’***), DMSO (***B’***), and PAP4 (***C’***) groups. Quantification of CMAP amplitudes showed no differences among groups or between ipsilateral and contralateral hemidiaphragm (***D***). Representative confocal *z*-stack image showing labeling of the diaphragm NMJ for the pan-axonal marker SMI-312 (green; ***F***, ***H***), the synaptic vesicle marker SV2 (green; ***F***, ***H***), and the marker of postsynaptic nicotinic acetylcholine receptors α-bungarotoxin (red; ***G***, ***H***). Nearly all NMJs in the ipsilateral hemidiaphragm remained fully innervated at eight weeks after hemisection; scale bar 50 μm. Quantification of the dorsal, medial, and ventral portions of the hemidiaphragm showed no differences in the percentage of fully-innervated, partially-denervated, or completely-denervated NMJs between the DMSO and PAP4 groups (***E***); *n* = 3 and *n* = 4 rats for DMSO and PAP4, respectively; 16 tissue sections per animal analyzed.

To evaluate functional innervation of the hemidiaphragm, we performed nerve conduction studies in which we supra-maximally stimulated the phrenic nerve with a transcutaneous needle electrode and recorded the CMAP response across the entire ipsilateral hemidiaphragm with a surface electrode. Representative CMAP traces show no differences in CMAP amplitude among uninjured control (ipsilateral: 7.1 ± 0.3 mV; contralateral: 6.9 ± 0.2 mV; Fig. 4*A*,*A’*), DMSO-only hemisection (ipsilateral: 6.7 ± 0.5 mV; contralateral: 7.0 ± 0.3 mV; Fig. 4*B*,*B’*), and PAP4-treated hemisection (ipsilateral: 7.8 ± 0.3 mV; contralateral: 7.7 ± 0.5 mV; Fig. 4*C*,*C’*) rats in the ipsilateral hemidiaphragm (*F*_(2,30)_ = 0.35; Tukey’s *post hoc* test; intact vs DMSO: *p* = 0.95; intact vs PAP4: *p* = 0.75; DMSO vs PAP4: *p* = 0.25; ANOVA; *n* = 6 rats per group; Fig. 4*D*). In addition, we found no differences in CMAP amplitudes obtained from hemidiaphragm ipsilateral and contralateral to the injury site in all groups (*F*_(1,30)_ = 0.01; Tukey’s *post hoc* test; intact: *p* = 0.99; DMSO: *p* = 0.97; PAP4: *p* > 0.99; ANOVA; *n* = 6 rats per group; Fig. 4*A–D*).

To quantify morphologic innervation, we performed immunolabeling of whole-mount hemidiaphragm muscle. We used SMI-312/neurofilament and synaptic vesicle protein 2 (SV2) antibodies to label phrenic motor axons all the way to their presynaptic terminals (Fig. 4*F*,*H*), and we labeled postsynaptic nicotinic acetylcholine receptors in the muscle with Alexa-conjugated α-bungarotoxin (Fig. 4*G*,*H*; Wright and Son, 2007; Wright et al., 2007, 2014). Confocal acquisition of *z*-stack projections revealed intact innervation of nearly all NMJs in both the DMSO-only (ventral: intact = 85.1 ± 8.3%; partial denervation = 4.8 ± 4.8%; complete denervation = 10.1 ± 5.1%; medial intact = 81.9 ± 9.1%; partial denervation = 7.2 ± 4.9%, complete denervation = 10.8 ± 5.8%; dorsal: intact = 98.4 ± 1.5%; partial denervation = 0.76 ± 0.76%, complete denervation = 0.7 ± 0.8%; *n* = 3 rats per group for all analyses) and PAP4 (ventral: intact = 96.2 ± 1.9, partial denervation = 3.8 ± 1.9; complete denervation = 0.0 ± 0.0; medial: intact = 88.6 ± 1.1%; partial denervation = 8.0 ± 0.7%; complete denervation = 3.4 ± 0.4%; dorsal: intact = 94.6 ± 3.2%; partial denervation = 1.9 ± 1.9%; complete denervation = 3.5 ± 2.2%; *n* = 3–4 rats per group for all analyses) groups (Fig. 4*H*); 90–100% of all NMJs were fully-innervated in all animals. Following quantitative analysis, we found no differences between DMSO-only and PAP4 in the percentage of NMJs in each subregion of ipsilateral hemidiaphragm that were intact (*F*_(2,14)_ = 1.25; Tukey’s *post hoc* test; dorsal: *p* = 0.97; medial: *p* = 0.78; ventral subregion: *p* = 0.34; ANOVA), partially-denervated (*F*_(2,14)_ = 0.08; Tukey’s *post hoc* test; dorsal: *p* > 0.99; medial: *p* > 0.99; ventral subregion: *p* > 0.99; ANOVA) or completely-denervated (*F*_(2,14)_ = 2.52; Tukey’s *post hoc* test; dorsal: *p* = 0.99; medial: *p* = 0.69; ventral subregion: *p* = 0.44; ANOVA; Fig. 4*E*).

Collectively, these data demonstrate that C2 hemisection resulted in no functional or morphologic denervation of the diaphragm by PhMNs and that PAP4 had no effect on diaphragm innervation compared to DMSO control.

### PAP4 promoted rVRG axon regeneration through the lesion and into caudal spinal cord

**Figure 5.**
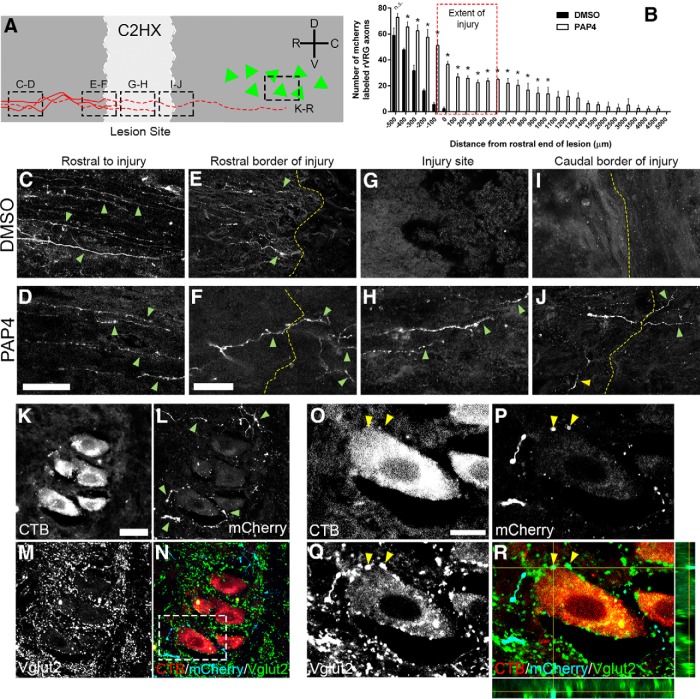
Ipsilateral rVRG axons regenerated through the lesion and formed putative synaptic connections with PhMNs. At eight weeks after injury, sagittal sections of the cervical spinal cord were examined for growth of mCherry-labeled axons originating in the ipsilateral rVRG at multiple locations relative to the lesion (***A***). Dotted boxes denote locations of images in subsequent panels, red lines represent rVRG axons, and green triangles represent PhMNs. Representative images of the lesion site reveal mCherry-labeled rVRG fibers were able to regenerate into and through the lesion and then back into the intact caudal spinal cord in PAP4-treated rats (***D***, ***F***, ***H***, ***J***), but not the DMSO-only controls (***C***, ***E***, ***G***, ***I***); scale bars: 100 μm (***C***, ***D***), 40 μm (***E–J***). Quantification of mCherry-labeled rVRG axon growth in 100 μm rostral-to-caudal bins using the rostral end of the lesion as the starting point (***B***). Confocal imaging show that regenerating mCherry+ rVRG axons (***L***, ***N***, ***P***, ***R***; arrowheads in ***L***) formed putative VGLUT2+ excitatory synaptic connections (***M***, ***N***, ***Q***, ***R***) with CTB+ PhMNs retrogradely-labeled from the ipsilateral hemidiaphragm (***K***, ***N***, ***O***, ***R***); scale bars: 50 μm (***K–N***), 30 μm (***O–R***). Orthogonal projection shows mCherry+/VGLUT2+ excitatory rVRG axon terminals located directly presynaptic to the soma of CTB+ PhMNs in the C3 spinal cord (***R***; arrowheads in ***O–R***). Dotted box in panel ***N*** denotes location of panels ***O–R***. No mCherry-labeled rVRG fibers were found at all in the ipsilateral PhMN pool of DMSO-treated controls (data not shown). *p < 0.05.

To determine effects of PAP4 on the growth response of damaged rVRG axons, we injected AAV2-mCherry into rVRG ipsilateral to hemisection. Specific locations of where images were obtained are diagramed in Figure 5*A*. Treatment with DMSO-only resulted in no regeneration at all into the lesion site (Fig. 5*E*,*G*) or through the lesion back into intact caudal spinal cord (Fig. 5*G*,*I*), coinciding with our previous work showing that injured rVRG axons do not regenerate following SCI (Urban et al., 2018). On the contrary, PAP4 induced robust long-distance regeneration of ipsilateral rVRG axons into the lesion (Fig. 5*F*,*H*), through the caudal lesion-intact interface (Fig. 5*J*) and back to the PhMNs (Fig. 5*K*,*L*). In sagittal sections, we quantified the total number of mCherry-labeled rVRG fibers in individual 100μm-wide bins starting at the rostral end of the lesion and proceeding in both rostral and caudal directions (Fig. 5*B*; Urban et al., 2018). Quantification of this regeneration shows that mCherry-labeled rVRG axons regenerated along the cervical spinal cord up to at least 4.5 mm from the distal lesion-intact interface (i.e., regeneration to the caudal portion of the PhMN pool), although we observed the greatest degree of regeneration closer to the injury at C3 (*F*_(1,5)_ = 38.5 Tukey’s *post hoc* test; ANOVA; *n* = 3 and *n* = 4 rats for DMSO and PAP4, respectively; 16 tissue sections per animal analyzed). We found no differences between the DMSO and PAP4 groups in number of mCherry-labeled axons at any distance ≥500 μm rostral to the lesion, suggesting equivalent rVRG neuron labeling with our AAV2-mCherry injections in the two groups.

### Regenerating rVRG axons formed putative synaptic connections with PhMNs

We next evaluated whether the regenerating rVRG axons made putative synaptic connections with denervated PhMNs ipsilateral to the injury. We performed triple-labeling with the excitatory presynaptic marker VGLUT2, DsRed (to enhance the mCherry signal) and CTB. We assessed the number of putative synaptic connections specifically between mCherry-positive rVRG fibers and CTB-labeled PhMN cell bodies at level C3 using confocal acquisition of *z*-stacks and quantification of rVRG axon-PhMN contacts using single-Z section analysis to establish direct apposition of presynaptic VGLUT2+/mCherry+ axon terminals and postsynaptic CTB+ PhMNs (Urban et al., 2018). Compared to DMSO-only that showed no putative rVRG-PhMN synaptic connection at all (0.0 ± 0.0 connections per CTB+ PhMN; images not shown), PAP4-treated animals showed significant synaptic reinnervation of PhMNs located ipsilateral/caudal to the lesion by regenerating rVRG axons (1.9 ± 0.6 connections per CTB+ PhMN; Fig. 5*K–N*,*O–R*); this quantification likely significantly underestimates the degree of synaptic connections given that CTB only marks the cell body and proximal dendrites of PhMNs and not the more distal dendritic processes. These findings demonstrate that, not only did injured rVRG axons undergo long-distance regeneration across the cervical SCI site, they importantly partially reconnected the original circuit by forming synaptic contacts with their PhMN targets.

### Sprouting of contralateral rVRG fibers

**Figure 6.**
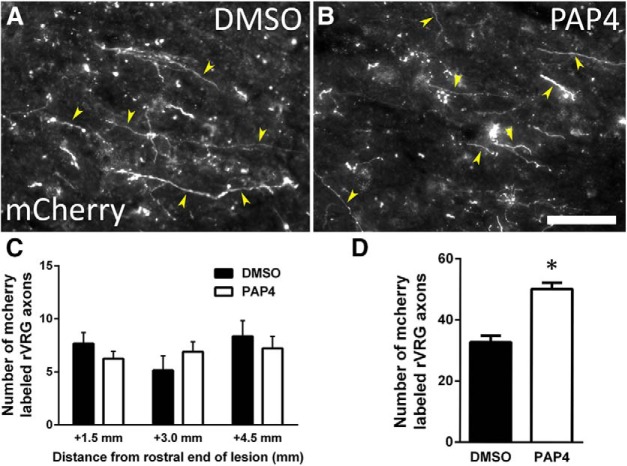
Sprouting of spared fibers originating in the contralateral rVRG. At eight weeks after injury, sagittal sections of the cervical spinal cord were examined for growth of mCherry-labeled axons originating in the contralateral rVRG at level C3, C4, and C5 (***A***). Representative images show no difference between DMSO control (***A***) and PAP4 (***B***) in the density of mCherry-labeled rVRG fibers in the C3 ventral horn ipsilateral to the lesion; yellow arrowheads in ***A***, ***B*** denote mCherry+ rVRG axons; scale bar: 100 μm. Quantification of the number of contralateral-originating rVRG fibers per area present within the ipsilateral ventral horn at levels C3–C5 (***C***). Distances from the lesion correspond to spinal cord levels: +1.5 mm to C3 (***A***, ***B***), +3.0 mm to C4, +4.5 mm to C5; *n* = 4 rats per group; *n* = 12 tissue sections analyzed per animal. PAP4 treatment increased the number of mCherry-labeled axons originating in the contralateral rVRG within the contralateral C3 ventral horn (***D***); *n* = 3 rats per group; nine tissue sections analyzed per animal. *p < 0.05.

We also explored modes of anatomic plasticity that could potentially underlie functional recovery besides regeneration of injured ipsilateral rVRG axons (Goshgarian et al., 1991; Boulenguez et al., 2007). Most human SCI cases are anatomically-incomplete (Ahuja et al., 2017), providing a substrate of spared axons that can sprout to form novel connections for reinnervating neuronal populations distal to a spinal cord lesion. In a separate cohort of animals, we labeled axons spared by the injury originating in the contralateral rVRG with AAV2-mCherry. In the ipsilateral ventral horn at locations caudal to the lesion, we assessed the total number of mCherry-labeled axon profiles within the CTB-labeled PhMN pool at 3 regions that correspond to C3 (1.5 mm from the caudal end of the lesion), C4 (3.0 mm), and C5 (4.5 mm) levels. Even in intact conditions and following C2 hemisection alone (i.e., no treatment), we found contralateral-originating rVRG axonal input to the PhMNs (data not shown), although levels were lower than input coming from the ipsilateral rVRG. We found no significant differences in total number of mCherry-labeled rVRG axon profiles between DMSO-only (C3: 7.7 ± 1.0 axon profiles; C4: 5.1 ± 1.4; C5: 8.4 ± 1.5; Fig. 6*B*,*C*) and PAP4 (C3: 6.2 ± 0.7 axon profiles; C4: 6.9 ± 1.0; C5: 7.2 ± 1.1; Fig. 6*A*,*C*) at any of these three distances (*F*_(1,18)_ = 0.33; Tukey’s *post hoc* test; C3: *p* = 0.06; C4: *p* = 0.08; C5: *p* = 0.27; *t* test; *n* = 4 rats per group; 12 tissue sections analyzed per animal; Fig. 6*C*), suggesting that increased drive from spared contralateral rVRG to ipsilateral PhMNs was likely not responsible for recovery. PAP4 treatment increased the number of mCherry-labeled axons originating in the contralateral rVRG within the contralateral C3 ventral horn (DMSO: 32.8 ± 2.1 axon profiles; PAP4: 50.2 ± 2.0 axon profiles; *n* = 9; *p* < 0.0001, *t* test; Fig. 6*D*), demonstrating that, while PAP4 did not have effects on these axons within the ipsilateral PhMN pool, systemic delivery of a PTEN inhibitor can stimulate plasticity of spared bulbospinal axon populations in the spinal cord.

### PAP4 promoted 5-HT axon growth and synaptic innervation of PhMNs caudal to the lesion

**Figure 7.**
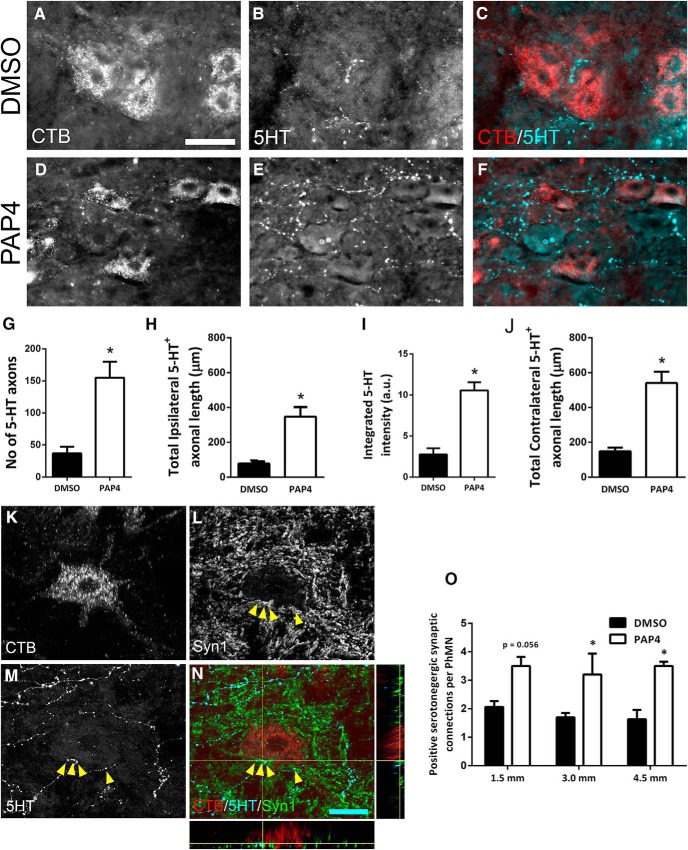
PAP4 promoted 5-HT axon growth and synaptic innervation of PhMNs caudal to the lesion. Compared to DMSO-only (***A–C***), PAP4 (***D–F***) enhanced the density of serotonergic axons directly surrounding CTB-labeled PhMNs at levels C3–C5; scale bar: 100 μm. Quantification demonstrates that PAP4 treatment increased the number of 5-HT+ axonal profiles (***G***), total length of 5-HT+ axonal profiles (***H***), and the integrated intensity of 5-HT immunostaining (***I***) compared to DMSO. PAP4 also increased the number of 5-HT+ axon profiles surrounding CTB-labeled PhMNs in the contralateral spinal cord (***J***). We also assessed the number of putative synaptophysin+ (***L***) synaptic connections between 5-HT axons (***M***) and CTB-labeled PhMNs (***K***) at levels C3–C5 using single-Z section analysis to establish direct apposition of presynaptic 5-HT+/synaptophysin+ axon terminals and postsynaptic CTB+ PhMNs (***N***). Compared to DMSO-only, PAP4 animals significantly increased the number of putative synaptic contacts between 5-HT axons and CTB-labeled PhMNs in the C3–C5 spinal cord ipsilateral to the hemisection (***O***). *p < 0.05.

Descending serotonergic input plays an important role in regulating the excitability of spinal cord motor neurons, in particular their response to glutamatergic signaling (Perrier et al., 2013). In the context of diaphragm function, PAP4 may be able to strengthen rhythmic excitatory input to PhMNs from spared and/or regrowing rVRG axons by increasing serotonergic innervation of PhMNs via 5-HT fiber sprouting (Mitchell et al., 1992; Zhou et al., 2001; Mantilla et al., 2012). To address this possibility, we quantified 5-HT immunostaining in the C3–C5 ventral horn ipsilateral to the C2 hemisection (Ghosh et al., 2018). Compared to DMSO-only (Fig. 7*A–C*), we found that PAP4 (Fig. 7*D–F*) enhanced the density of serotonergic axons directly surrounding CTB-labeled PhMNs at levels C3–C5. We conducted this analysis of 5-HT fiber growth within the CTB-labeled PhMN pool by performing quantification of (1) numbers of 5-HT+ axon profiles, (2) length of these 5-HT+ axon profiles, and (3) integrated intensity of 5-HT immunostaining (Ghosh et al., 2018). Compared to DMSO-only, PAP4 treatment increased the number of 5-HT+ axonal profiles (DMSO: 37.0 ± 10.0 axon profiles; PAP4: 155.0 ± 24.9 profiles, *t* test, *p* < 0.001; Fig. 7*G*) and total 5-HT+ axonal length (DMSO: 78.9 ± 17.7 μm; PAP4: 347.5 ± 54.5 μm, *t* test, *p* < 0.001; Fig. 7*H*). In addition, PAP4 increased integrated intensity of 5-HT immunostaining compared to DMSO-only (DMSO: 2.8 ± 0.7 a.u.; PAP4: 10.6 ± 0.98 a.u; *t* test, *t* test; *p* < 0.001; Fig. 7*I*). PAP4 treatment also stimulated serotonergic axon sprouting in the contralateral PhMN pool, demonstrating that effects of PAP4 on 5-HT axon plasticity were not restricted to only the side of injury (DMSO: 148.0 ± 21.1 profiles; PAP4: 540.7 ± 64.7 profiles; *n* = 3; *p* = 0.005, *t* test; Fig. 7*J*).

Lastly, we conducted quantitative analysis of synaptic input to ipsilateral PhMNs by serotonergic axons using multi-labeling immunohistochemistry for 5-HT+ axons, CTB+ postsynaptic PhMNs and the presynaptic marker synaptophysin. We assessed the number of putative synaptic connections between 5-HT fibers and CTB-labeled PhMNs at levels C3–C5 using confocal acquisition of *z*-stacks and quantification of 5-HT axon-PhMN contacts using single-Z section analysis to establish direct apposition of presynaptic 5-HT+/synaptophysin+ axon terminals and postsynaptic CTB+ PhMNs (Ghosh et al., 2018; Fig. 7*K–O*). Compared to DMSO-only (C3: 2.1 ± 0.2, C4: 1.7 ± 0.2, C5: 1.6 ± 0.3 contacts per PhMN soma), PAP4 animals (C3: 3.5 ± 0.3, C4: 3.2 ± 0.7, C5: 3.5 ± 0.2 contacts per PhMN cell body) showed significantly more putative synaptic contacts between 5-HT axons and PhMNs (C3: *p* = 0.056, C4: *p* = 0.045, C5: *p* = 0.013; unpaired *t* test; *n* = 3 rats per group for all analyses; Fig. 7*N*).

### Effects of re-lesion on PAP4-induced restoration of diaphragm function

**Figure 8.**
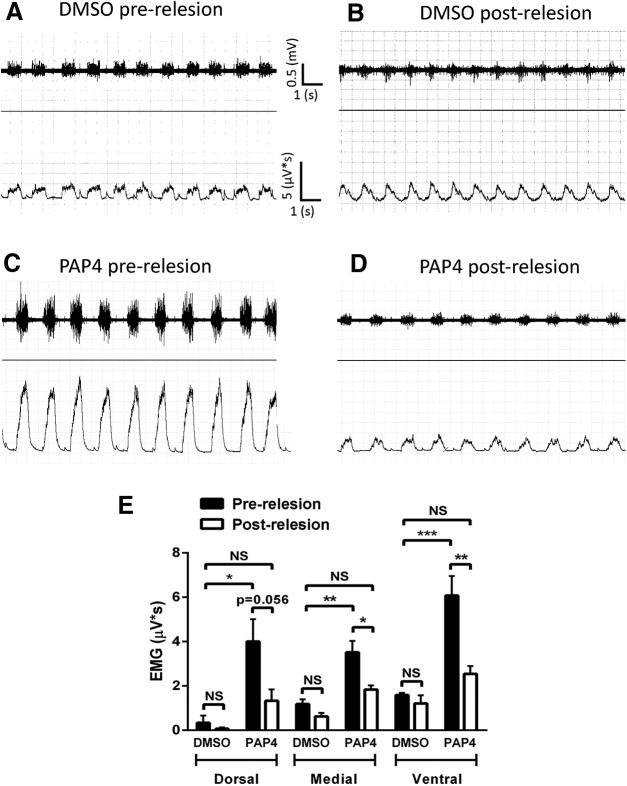
Effects of re-lesion on PAP4-induced restoration of diaphragm function. At eight weeks after hemisection, we performed a re-lesion through the C2 injury site of both DMSO-only and PAP4 groups. Representative traces of EMG recordings from the ventral hemidiaphragm show no change in EMG amplitude recordings between DMSO pre-re-lesion and DMSO post-re-lesion groups (***A***, ***B***). There was a significant loss of inspiratory burst amplitude following the re-lesion (***D***) in the PAP-treated group compared to the recordings immediately before re-lesion in the same animal (**C**). Quantification of inspiratory EMG bursts in the dorsal, medial and ventral subregions of the ipsilateral hemidiaphragm before and after re-lesion (***E***). *p < 0.05. **p < 0.01. ***p < 0.001. ns, not significant *p* ≥ 0.05.

Given the robust degree of rVRG axon regeneration and rVRG-PhMN synaptic reconnectivity we observed following PAP4 administration, we sought to determine whether regeneration was causally responsible for the resulting recovery of diaphragm function. Again at eight weeks after injury, we conducted EMG recordings both before (dorsal: DMSO: 0.3 ± 0.3 mV/s, *n* = 3; PAP4: 4.0 ± 1.0, *n* = 4; medial: DMSO: 1.2 ± 0.2, *n* = 3; PAP4: 3.5 ± 0.5, *n* = 4; ventral: DMSO: 1.6 ± 0.1, *n* = 3; PAP4: 6.1 ± 0.9, *n* = 3) and after (dorsal: DMSO: 0.1 ± 0.1 mV/s, *n* = 3; PAP4: 1.3 ± 0.5, *n* = 4; medial: DMSO: 0.6 ± 0.2, *n* = 3; PAP4: 1.8 ± 0.2, *n* = 4; ventral: DMSO: 1.2 ± 0.4, *n* = 3; PAP4: 2.6 ± 0.4, *n* = 3) a surgical re-lesion through the center of the injury site in a subset of animals (Fig. 8*A–D*). Re-lesion had no effect on the small degree of recovery observed with DMSO (*F*_(1,12)_ = 4.40; Tukey’s *post hoc* test; dorsal: *p* > 0.99; medial: *p* > 0.99; ventral: *p* > 0.99; ANOVA; Fig. 8*A*,*B*,*E*); this modest functional improvement is normally observed after C2 hemisection and is likely driven by spared contralateral rVRG input that is present even in intact conditions (Goshgarian et al., 1991; Boulenguez et al., 2007). On the contrary, PAP4-stimulated recovery was lost with re-lesion at all three subregions of the ipsilateral hemidiaphragm (Fig. 8*C*,*D*,*E*). Representative traces from the ventral hemidiaphragm following PAP4 administration show inspiratory bursts in the same animal both immediately before and after re-lesion (Fig. 8*A–D*). EMG burst amplitudes after re-lesion of PAP4 rats were significantly reduced compared to the same PAP4-treated animals before re-lesion (*F*_(1,16)_ = 24.54; Tukey’s *post hoc* test; dorsal: *p* = 0.02; medial: *p* = 0.41; ventral: *p* = 0.006; ANOVA) and were not significantly different from C2 hemisection animals treated with DMSO-only (*F*_(5,28)_ = 3.07; Tukey’s *post hoc* test; dorsal: *p* = 0.97; medial: *p* = 0.99; ventral: *p* = 0.98; ANOVA). Importantly, this loss of EMG amplitude in the PAP4 animals was not due to a spinal shock effect. In a separate study with C2 hemisection rats, we also obtained significant recovery of ipsilateral hemidiaphragm EMG amplitudes using a different experimental manipulation (i.e., PTP-sigma inhibitory peptide) that did not promote any axon regeneration through the lesion. Unlike in the current study with PAP4, re-lesion of these animals had no effect at all on EMG recovery in the ipsilateral hemidiaphragm (i.e., the recovery was completely maintained after re-lesion; data not shown), demonstrating that the re-lesion procedure itself does not non-specifically compromise EMG amplitude.

## Discussion

Promoting the reconstruction of rVRG-PhMN-diaphragm circuitry is a potentially-powerful strategy for addressing respiratory dysfunction after cervical SCI. Therapies aimed at achieving synaptic reconnection of rVRG axons with denervated PhMNs after SCI have been relatively unsuccessful to date. A small number of studies have begun to show the utility of promoting regrowth of descending axonal input to the PhMN pool in SCI models (Charsar et al., 2017). For example, removing the inhibitory glycosaminoglycan side chains from chondroitin sulfate proteoglycans (along with a peripheral nerve graft) promotes growth of serotonergic axons that regulate PhMN excitability (Alilain et al., 2011). Our data suggest this PAP4 approach has profound clinical relevance both for understanding circuit plasticity and for eventually developing therapies. A number of studies have supported that PTEN is a major contributor to reduced intrinsic growth capacity of mature neurons. Although the capacity for CNS axon regeneration diminishes with age (Geoffroy et al., 2016), previous work has shown that adult neurons are able to regenerate through modulation of PTEN activity following injury using selective deletion of PTEN via several methods, including Cre KO mice (Park et al., 2008; Sun et al., 2011; Geoffroy et al., 2015), shRNA vectors (Lewandowski and Steward, 2014) and pharmacological inhibition (Ohtake et al., 2014). We achieve a significant degree of regeneration of injured rVRG axons with systemic delivery of PAP4 alone; rVRG axons regrow through the C2 hemisection lesion and back toward a large extent of the denervated PhMN pool (up to two to three segments caudal to injury), although the majority of regenerating rVRG axons only reach the C3 portion. Importantly, this regeneration is associated with robust recovery of diaphragm function.

Given that systemic delivery could potentially elicit unwanted side effects, we injected PAP4 for only a restricted time, yet still achieved significant rVRG axon regeneration. Furthermore, we are following up this work by reducing delivery duration to determine the minimal window that successfully promotes therapeutic effects. Similarly, it will be important to determine whether delayed delivery (including in the setting of chronic injury) can induce rVRG regeneration and diaphragmatic recovery, particularly as PTEN inhibition may not be capable of promoting such a robust axon growth response, for example, after a mature glial scar has already formed, at longer time points following axonal injury and/or in older animals (Steinmetz et al., 2005; Ylera et al., 2009; Geoffroy et al., 2016).

The degree of improvement after PAP4 administration differed across the hemidiaphragm; functional EMG recovery was greatest in the ventral diaphragm subregion, which receives innervation primarily from the most rostral PhMNs. Furthermore, regeneration of ipsilateral rVRG axons induced by PAP4 was greatest at C3 (and progressively decreased with caudal distance from the lesion). This suggests that rVRG regeneration may have played a particularly important role in recovery for the ventral subregion because of differential rVRG axon plasticity responses across the PhMN pool. It is possible that the greater levels of rVRG axon regeneration and PhMN reinnervation by these fibers that occurred at the most rostral PhMN locations (combined with the robust 5-HT sprouting at this rostral location) resulted in the enhanced recovery observed in the ventral subregion, while the reduced (but still significant) recovery observed at the medial and dorsal subregions was due to the fact that only 5-HT axon sprouting occurred at the most caudal portions of the PhMN pool. It will be important in future work to perform time-course analysis of rVRG axonal growth along the cervical spinal cord and to correlate this process with the timing of EMG recovery in the different diaphragm subregions; this would be greatly aided by technologies such as repeated recording from the same animal using chronically-implanted telemetric electrodes (Mantilla et al., 2011; Bezdudnaya et al., 2018).

With this work, we sought to determine which modes of rVRG-PhMN circuit plasticity can drive recovery of respiratory function following cervical SCI. Importantly, in SCI and other types of CNS trauma, we do not understand which forms of circuit re-connectivity are truly able to promote recovery, which significantly limits our ability to develop targeted therapies in an informed manner. This is the case for respiratory function, but also for the other circuits and functional outcomes affected by nervous system trauma. To address this critical circuit mechanism issue, we show that PAP4 was able to promote robust levels of rVRG axon regeneration; when regeneration was surgically ablated, recovery was completely lost, demonstrating that regeneration through the injury was responsible for driving functional improvement.

We observed differences in the degree of rVRG axon regeneration across the ipsilateral PhMN pool (i.e., that relatively little regeneration occurred into the more caudal portions of the PhMN pool). These data may suggest that, while the observed rVRG axon regeneration and synaptic reconnectivity with PhMNs is exciting, this mode of circuit plasticity may actually not be the mechanism underlying functional recovery induced by PAP4. Furthermore, this mechanism may be at play, but only for the recovery of ventral diaphragm function given the significantly greater rVRG axon regeneration occurring at the most rostral PhMN pool locations. Serotonergic axon growth may instead be the plasticity mode operating across the PhMN pool to drive recovery at all three diaphragm subregions given that we observed equivalent levels of 5-HT axon synaptic innervation of PhMNs at all three caudal distances from the lesion. It is also possible that both rVRG and serotonergic axon plasticity is contributing to functional recovery, but that the relative contribution of each may vary significantly across the PhMN pool.

In addition to observing relatively low amounts of rVRG axon regeneration caudally past C3, we also did not achieve robust levels of synaptic reinnervation of PhMNs by rVRG axons, even at C3 where we observed the greatest amount of regeneration. This further suggests that rVRG axon regeneration may not be playing a role in functional recovery at medial and dorsal diaphragm subregions and may only be playing a partial role at the ventral subregion (or may not even be involved here as well). Although we did not promote a large amount of monosynaptic connectivity between regenerating rVRG axons and PhMNs, it is possible that these regenerating rVRG axons still contributed to functional recovery, including at all three muscle subregions. Re-lesion completely ablated PAP-induced EMG recovery, demonstrating that regeneration (of some axon population) through the lesion was responsible for driving recovery and suggesting that rVRG axons were at least partially involved in this effect. As we found relatively low amounts of rVRG axon regeneration caudally past C3, it is possible that the regenerating rVRG axons that reached the C3 spinal cord innervated intrasegmental and intersegmental interneuron populations that allow these regrowing rVRG axons to also mediate inspiratory activation of more caudally-located PhMNs in the C4–C5 spinal cord via circuits that are not restricted to rVRG-PhMN monosynaptic connection. Cervical SCI induces plasticity of rVRG connections with pre-phrenic cervical interneurons (Lane et al., 2008b). In future studies, it will be important to assess synaptic input of ipsilateral-originating rVRG axons and contralateral-originating rVRG axons onto spinal pre-phrenic interneurons to determine whether PTEN manipulation promoted recovery beyond just mono-synaptic circuitry.

Given the important role of descending serotonergic input in modulating PhMN excitability (Dale-Nagle et al., 2010), enhanced 5-HT fiber growth (alone or in combination with rVRG axon regeneration) may have been the mechanism underlying respiratory recovery. To this notion, we found that PAP4 increased 5-HT fiber density in the C3–C5 ventral horn caudal to C2 hemisection around the PhMN pool; we also found that PAP4 significantly enhanced putative synaptic connections of these serotonergic axons specifically with PhMNs. We found serotonergic axons within the lesion site in both DMSO-control and PAP4 animals (data not shown), suggesting that the re-lesion could have resulted in the loss of diaphragm functional recovery by severing these regrowing 5-HT axons (and not by damaging the regenerating rVRG axons; or a combination of both). However, the degree of putative synaptic innervation of CTB-labeled PhMNs by 5-HT axons (shown in Fig. 7*K–O*) was equal across the entire PhMN pool, suggesting that this serotonergic axon input to the PhMNs on the side of SCI was not due to regeneration of serotonergic axons through the lesion but instead was due to local growth of axons spared by the hemisection already present at these various C3–C5 locations. Otherwise, we would expect to find the greatest degree of serotonergic axon-PhMN synaptic connection at C3 (i.e., the most rostral site closest to the injury), with decreasing levels of serotonergic axon-PhMN connections at farther caudal distances (i.e., similar to pattern of rVRG axon counts at C3–C5 shown in Fig. 5*B*). To further address this issue, in future work we will use chemogenetic approaches (e.g., DREADDs; Roth, 2016) to selectively silence ipsilateral or contralateral rVRG neurons or serotonergic raphe neurons to more definitively determine which neuronal population(s) underlie functional recovery in response to PAP4 and other interventions.

Given that PTEN manipulation has previously been shown to promote sprouting of other neuron populations such as corticospinal tract axons (Liu et al., 2010), it is interesting that PAP4 did not stimulate contralateral rVRG sprouting in the ipsilateral ventral horn in our study. As one explanation, it is possible that uninjured contralateral rVRG neurons have insufficient PTEN activity levels after SCI to limit a sprouting response and therefore PAP4 would be unable to further induce a response from these spared axons. While our data do not show morphologic effects of PAP4 on sprouting of these fibers within the PhMN pool ipsilateral to the hemisection, they may still be playing a role in the effects of PAP4 by, for example, altering synaptic connections between these rVRG axons and PhMNs via very short-distance sprouting that we cannot appreciate with our analysis. We have not experimentally addressed this issue; therefore, future studies will be necessary to further explore the contribution of spared rVRG input to the actions of PAP4.

Although PTEN is thought of primarily in the context of CNS injury as a neuronal-intrinsic inhibitor of axonal growth potential (Park et al., 2010), PAP4 may have stimulated rVRG axon regeneration through a non-cell autonomous mechanism. For example, PAP4 may have altered properties of the glial and/or fibrotic scar (Goncalves et al., 2015; Chen et al., 2016) such that cellular components of these scar structures produced increased pro-growth factors or had reduced synthesis of inhibitory molecules. While we did not observe any quantitative difference in overall morphology of the astrocyte scar (i.e., size of the scar), it is not possible to examine every possible effect and therefore completely rule this out. We did find that PAP4 increased signaling along the mTOR axis specifically in rVRG neurons, supporting that the effect of PAP4 on rVRG axon plasticity was at least in part due to an increase in neuronal-intrinsic axon growth potential. We also performed immunohistochemistry on medulla and cervical spinal cord by double-labeling for pS6K and the astrocyte lineage marker GFAP; we found no effect of PAP4 on pS6K levels in astrocytes in-or-around the lesion site or in the rVRG (data not shown), suggesting that PAP4 was acting through a more direct effect on neurons (although more thorough analysis would be required to more strongly substantiate such a claim). We also previously showed using a biotintylated version of PAP that the antagonist peptide does get into neurons following injection into SCI rats (Ohtake et al., 2014). Although we have not performed the same analysis again in the current study, our previous work suggests that PAP4 does get into rVRG neurons and that PAP4 is exerting its effect at least in part directly on rVRG neurons.

While we have shown that PAP4 promoted significant recovery of diaphragm function *in vivo* under eupnic breathing conditions, there are limitations of our functional analysis. We conducted electrophysiological recordings (i.e., EMGs and CMAPs) to specifically assess effects on diaphragm activation and PhMN-diaphragm circuitry, although EMGs and CMAPs do not fully provide evaluation of the physiologic effects on respiratory function. Importantly, we did not perform assessments of overall ventilatory function such as whole-body plethysmography or blood gas measurements. In addition, we did not conduct EMG recordings in response to hypercapnic and hypoxic challenges. To further examine the functional effects of our PAP4 approach on respiratory circuitry, in future work, we will need to test effects on these additional functional outcomes and in response to respiratory challenges.

In conclusion, we report that non-invasive systemic delivery of a peptide that inhibits PTEN function results in regeneration of rVRG axons that are critical to diaphragmatic respiratory function and that are damaged by cervical SCI. These regenerating rVRG axons extend along the PhMN pool to synaptically reconnect with their appropriate targets. However, PAP4 did not stimulate sprouting of axons originating in the contralateral rVRG or increased synaptic input of these fibers to denervated PhMNs, suggesting that plasticity of spared rVRG axonal populations was not responsible for driving improvement. In addition, PAP4 resulted in significant 5-HT axon growth and synaptic innervation of PhMNs caudal to the lesion, suggesting that plasticity of both rVRG and 5-HT axon populations may have acted in concert to drive recovery. Finally, our findings demonstrate that axon regeneration through the lesion is causally responsible for promoting the robust degree of restoration of diaphragm function induced by PAP4, although we did not conclusively determine the specific regenerating axon population(s) mediating this effect. This approach may have significant therapeutic implications for treating persistent debilitating respiratory dysfunction in individual affected by SCI.

## Synthesis

Reviewing Editor: Jeffery Twiss, University of South Carolina Decisions are customarily a result of the Reviewing Editor and the peer reviewers coming together and discussing their recommendations until a consensus is reached. When revisions are invited, a fact-based synthesis statement explaining their decision and outlining what is needed to prepare a revision will be listed below. The following reviewer(s) agreed to reveal their identity: Carlos Mantilla, John Houle. Note: If this manuscript was transferred from JNeurosci and a decision was made to accept the manuscript without peer review, a brief statement to this effect will instead be what is listed below.

This manuscript, which was previously reviewed in a submission to J Neurosci, addresses an important gap in the field of neuroplasticity post-SCI. Overall, the reviewers felt that many of the major comments from the previous review were addressed. However, they emphasized the limitations of correlating structure and function with the model used. There is uncertainty for whether the tracing experiments clearly demonstrate axons innervating/regenerating into distal regions of the ipsilateral phrenic nucleus, but there is clear return of significant motor function. The revised manuscript does not adequately present/discuss the possible reasons for that recovery. There seems to be some reluctance by the authors to acknowledge a substantial role for serotonergic axons in the diaphragm recovery seen here. There is no mention of a possible role of spinal interneuron plasticity and recovery of function, and this should be addressed. These issues are particularly important when the quantitative data for VRG axon number and distance traveled distal to the lesion is considered (see below) - these measures both seem rather small considering the extent of physiological recovery demonstrated. The reviewers and reviewing editor suggest that the authors give more consideration to serotonergic and contralateral VRG sprouting as a source for distal diaphragm recovery in their discussion. The differences/lack of differences across diaphragm regions are only superficially considered and should be more fully addressed. This information is important to the interpretation of axon regeneration.

Major and minor points from reviewers are synthesized below - though these seem extensive, the bulk could be addressed by text changes and re-analyses of existing data. The possibilities of tissues available for some of the additional analyses (e.g., spinal cords may have been imaged but not analyzed) and supporting data potentially excluded from the manuscript were raised in our online discussion.

Major points:

1. The extent of axon regeneration did not correspond through the full phrenic nucleus yet all parts of the diaphragm showed good recovery of function (though not acknowledged by the authors). Figure 5B shows fewer than 40 VRG axons enter the lesion zone, fewer than 30 exit the lesion zone, and fewer than 20 reach 500 um from the rostral end of the lesion zone. It appears that about 5 axons reach 1500 μm from the rostral end of the lesion zone, which the authors state is the distal end of C3. In the C3 cord the authors state there are 1.8 synapses observed per CTB- labeled PhMN but if there are only 5 or so axons that would mean maybe 10 synapses, which may not constitute significant synaptic reinnervation as is stated on page 18. The issue is the data is very interesting but somewhat over stated (i.e., not a large number of axons, not significant reinnervation, and not restoration of original circuitry, at least not solely due to VRG axons). The return of function was effectively lost when the original lesion site was re-lesioned. This is a crude but effective demonstration that a major source of recovered function was from axons growing through the lesion site. The authors recognize the need for more sensitive and cell specific approaches for future experiments. However, it is critical that serotonergic sprouting, both ipsilateral and contralateral, be given more consideration as a source of reinnervation/function that may or may not be abolished by a second lesion.

2. Measurements of eupneic diaphragm EMG amplitude were used to conclude that PAP4 treatment induced a greater extent of functional recovery 8 weeks post-injury, but deficits in other responses (e.g., following exposure to maximal chemoreceptor activation) or in ventilation were not measured. It is impressive that there is significant recovery of ventral region diaphragm activation (Figure 3E) that likely is attributed to having more axons reaching C3 than either C4 or C5. Though it is difficult to evaluate the importance of the small change primarily in one section of the diaphragm, the data show activity in the medial and dorsal diaphragm regions that is significantly better than DMSO treated animals but still considered to be no recovery compared to intact animals by the authors. Whether this is due to reduced number of VRG axons is not clear and not discussed. This warrants further discussion about distance of regeneration and assignment of functional activity based solely upon the re-lesion experiment. Also, several rat atlases indicate that the C3 segment of spinal cord is about 3.5 mm in length (same for C4 and C5), so there is some confusion here about what constitutes C3, C4 and C5 levels in this study.

3. Strong concluding statements regarding the causal relationship of rVRG axon regeneration to recovery following PAP4 treatment are still present in the Abstract, New & Noteworthy, and Introduction sections. Several interpretation issues raised in the previous and current reviews regarding the underlying mechanisms for the observations in this report are present. Strongly worded concluding statements in the Results are also not justified.

4. Changes in phospho-S6 kinase immunofluorescence 8 weeks after injury are interpreted as a direct consequence of PAP4 treatment weeks before, yet the mechanism is likely complex given that PAP4 treatment occurred weeks earlier, information regarding the pharmacology of PAP4 is limited, and treatment was systemic and not selective to one cell type (e.g., rVRG neurons vs. surrounding glia). These considerations limit the interpretation of underlying mechanism and were identified by previous reviewers. The authors have not sufficiently moderated their concluding statements throughout the manuscript and not just in the discussion (e.g., abstract, introduction, results sections suggest that the evidence provided in the manuscript is conclusive; e.g., page 5 - last paragraph; page 15 - end of 3rd paragraph).

5. Interpretation of the lack of effects of PAP4 treatment on glial scarring or injury severity is limited to a single averaged measurement of the maximal length of the GFAP scar in 3 animals/group. This seems insufficient to support the authors' conclusion. Do they have other data to support this conclusion or they could present these data as suggestive emphasizing additional experiments are needed for conclusive evidence.

6. Strongly worded concluding statements in the Results should be moderated in accordance with these considerations (e.g, page 17 - “PAP4 promoted robust, long-distance rVRG axon regeneration through the lesion and into caudal spinal cord” or page 20 - “Re-lesion ablated PAP4-induced restoration of diaphragm function”) .

7. Important information for the field is not consistently reported or considered. For example, ipsilateral and contralateral- originating rVRG axonal input to the PhMNs is simply not shown. Furthermore, this type of information may help inform whether rVRG axons cross the midline above or below C2 (e.g., at the level of the motor pool). Furthermore, information for Figures 5 and 6 on the contralateral phrenic motor pool is important to the interpretation of directed vs. generalized axonal sprouting and to provide confidence in the robustness of the methods (e.g., the density of mCherry axons on the side of rVRG injection proximal to the lesion should be similar, etc.).

8. Similar considerations for serotonergic innervation of the PhMN pool to those raised in point # 7 apply. Please, consider reporting of findings in the PhMN pool contralateral to injury.

9. The discussion section should reflect the limitations resulting from the methodological issues identified in this (and the previous) review. Concluding statements regarding the degree of functional loss (and restoration) in the C2 hemisection model should be revised in light of the available evidence regarding functional impairment. This point is particularly relevant to the present study given the very restricted set of functional measures used.

Minor points:

1. Analyses usually involve multiple groups and measurements within an animal, yet the statistical information provided does not sufficiently identify the comparisons being performed. Mostly post hoc p values are provided when there are multiple comparisons performed even within an animal (e.g., multiple sites, side of injury, level of the spinal cord, etc).

a) Please, report all measurements and p-values with consistent precision (e.g., one decimal point for lesion length measurements and average fluorescence intensity/cell - pages 15 and 20). Three decimal points for the proportion of NMJ types is clearly not justified (page 17). Statistical significance levels are also inconsistently presented throughout the Results section.

b) Please, indicate clearly and justify when measurements are not averaged within an animal (e.g., pS6k immunofluorescence was compared across groups using the number of cells, but significant differences across animals are likely to be present).

c) There is insufficient clarity regarding the analysis of diaphragm EMG activity (pg. 16; and, Fig. 3). Measurements were performed over 5 min across three sections on each side of the diaphragm for each animal, yet the F values provided do not clearly articulate what comparisons are being conducted; only post-hoc Tukey details are provided).

2. Please report other ventilatory parameters (e.g., respiratory rate & duty cycle both of which could be derived from the EMG). It is unfortunate that they seemingly did not perform additional functional studies (e.g., with maximal chemoreceptor stimulation). As a result, any functional improvement was incompletely studied and the present study is more promising that conclusive of its potential therapeutic role.

3. The Methods section could be shortened if measurements conducted with similar techniques were aggregated (e.g., immunohistochemical analyses). Several additional methodological issues deserve attention, some of which also detract from the confidence in the authors' interpretation of their findings.

a) Please, carefully review report of the timing of intramedullary AAV injection. The text (pg 15) and figure 1 legend are inconsistent (1 vs. 2 weeks post-injury.

b) Also, report for timing of CMAP measurements and intrapleural CTB injection are inconsistent (text pg 15 - week 7 post-injury vs. Figure 1 legend - 4 days before terminal = 8 weeks).

c) Measurements of synaptic contacts on neurons are presented as contacts/labeled motor neuron, yet there are likely differences in the motor neuron surface area that was examined. Accordingly, please provide indicate whether only motor neuron soma were examined and if there were considerations regarding the extent of membrane surface area studied.

d) Please, provide the orthogonal projections for Fig. 7M.

e) The representative tracings in Fig A-B do not appear to match the summary data for the ventral diaphragm DMSO group.

## Author Response

Response to reviewer comments: Urban and Ghosh, et al. - submission to eNeuro

1st May 2019

Dear Dr. Lepore:

I am pleased to inform you that your manuscript, “Long-distance axon regeneration promotes recovery of diaphragmatic respiratory function after spinal cord injury,” has been judged potentially suitable for publication in eNeuro, provided appropriate revisions are made. The decision was a result of the reviewers and me coming together and discussing our recommendations until a consensus was reached. I have put together a fact-based synthesis statement explaining the decision and appended it to this email.

It is important that you revise your manuscript to address the reviewers' concerns, and submit a point-by-point reply to the reviews that indicates your response to each concern. Before a final decision about publication is made, we will have your revision re-reviewed by one or both reviewers.

Your revision must include the manuscript with new text indicated in a bold or colored font to aid the re-review of the manuscript. Separately, please provide a clean copy of the manuscript that includes the title page which will be published if accepted. Please closely review your manuscript at this time for any final corrections in style or substance. If your revision is accepted, you will not be allowed to make style or content changes at the proof stage.

Overall response to Editor: We appreciate the helpful comments of the reviewers on our initial submission to eNeuro. We feel that the changes made based on the reviewers' input make for a greatly improved manuscript. We have enclosed a point-by-point set of responses to each reviewer comment, and we have made necessary changes to the text (which are highlighted in red) and figures, including the addition of a significant amount of new data requested by the reviewers.

At eNeuro we value the contributions of individual authors and want to offer the opportunity to better acknowledge the roles of all authors. As of December 2018, corresponding authors may include specific individuals' roles to data acquisition and analysis at the end of each figure legend. For more information, please see: http://www.eneuro.org/content/preparing-manuscript#fig_legends

Your submission must also include publication-quality figures, each in a separate EPS or TIFF (300 dpi) file, if applicable. Please make sure your figures adhere to the style requirements to avoid delays in manuscript processing. Detailed guidelines for figures are available in our Information for Authors found here: http://www.eneuro.org/content/information-authors

If you would like to include a visual abstract with your revision, you can refer to instructions found here: http://www.eneuro.org/content/preparing-manuscript#visual

When uploading the revised manuscript, there is a link available for the corresponding author to complete the electronic License to Publish form. A link to the form will also be available on the author's home page at http://eNeuro.msubmit.net if not completed at time of submission.

Please return your revision within 3 months of this decision. If you need more time, please contact eNeuro@SfN.org. A checklist for submitting a revision can be found in our Information for Authors here: http://www.eneuro.org/content/submitting-manuscript

When you are ready to submit your revision, you can log in using the link below and click on the manuscript number to create your revision. If you have any questions or concerns, please contact eNeuro@SfN.org.

https://eneuro.msubmit.net/cgi-bin/main.plex

Yours sincerely,

Jeffery Twiss

Reviewing Editor, eNeuro

Manuscript Instructions

- Remove the Abbreviations paragraph/list. Please only use nonstandard abbreviations if a term appears three or more times (the initial appearance of the term and one other time). Spell out the term at first occurrence and introduce the abbreviation by placing it in parentheses after the term. Units should conform to the International System of Units (SI) (see the International System of Units Brochure), except for temperatures, which should be expressed in degrees Celsius.

Click the following link for a list of some standard abbreviations:

http://eneuro.org/sites/default/files/Standard_Abbreviations_List.pdf

Response: We have removed the abbreviations list at the beginning of the manuscript, and we have correctly presented abbreviations in the text when appropriate.

---------------------------------------------

Synthesis of Reviews:

Computational Neuroscience Model Code Accessibility Comments for Author (Required):

N/A

Significance Statement Comments for Author (Required):

Appropriate

Comments on the Visual Abstract for Author (Required):

N/A

Synthesis Statement for Author (Required):

This manuscript, which was previously reviewed in a submission to J Neurosci, addresses an important gap in the field of neuroplasticity post-SCI. Overall, the reviewers felt that many of the major comments from the previous review were addressed. However, they emphasized the limitations of correlating structure and function with the model used. There is uncertainty for whether the tracing experiments clearly demonstrate axons innervating/regenerating into distal regions of the ipsilateral phrenic nucleus, but there is clear return of significant motor function. The revised manuscript does not adequately present/discuss the possible reasons for that recovery.

Response: We have now greatly expanded upon our discussion of the potential underlying neuroanatomical mechanisms that are driving functional recovery. Based on our data and the reviewers' excellent comments, we discuss the differences in the degree of rVRG axon regeneration across the ipsilateral PhMN pool (i.e. that relatively-little regeneration occurs into the more caudal portions of the PhMN pool). We now discuss how these data may suggest that - while the observed rVRG axon regeneration and synaptic reconnectivity with PhMNs is exciting - this mode of circuit plasticity may actually not be the mechanism underlying functional recovery induced by PAP4. Furthermore, we discuss how this mechanism may be at play, but only for the recovery of ventral diaphragm function given the significantly greater rVRG axon regeneration occurring at the most rostral PhMN pool locations. We also discuss how serotonergic axon growth may instead be the plasticity mode operating across the PhMN pool to drive recovery at all three diaphragm sub-regions given that we observed equivalent levels of 5-HT axon synaptic innervation of PhMNs at all three caudal distances from the lesion. Lastly, we state that it is possible that both rVRG and serotonergic axon plasticity is contributing to functional recovery, but that the relative contribution of each may vary significantly across the PhMN pool. Based on the reviewers' excellent comments, we feel that this discussion provides a much more nuanced interpretation of our findings and does not overstate the contribution of rVRG axon regeneration given the relatively-modest levels that we observe beyond approximately the first millimeter caudal to the lesion.

There seems to be some reluctance by the authors to acknowledge a substantial role for serotonergic axons in the diaphragm recovery seen here.

Response: As described above, we have modified our discussion; we now provide a more nuanced interpretation that highlights the likely contribution of serotonergic input and does not overstate the contribution of rVRG axon regeneration.

There is no mention of a possible role of spinal interneuron plasticity and recovery of function, and this should be addressed.

Response: We previously included brief mention of these interneurons that have been shown to play an important role in phrenic motor circuitry. We have now expanded on this. However, we have not experimentally assessed synaptic input of ipsilateral-originating rVRG axons, contralateral-originating rVRG axons or 5-HT axons onto spinal pre-phrenic interneurons, though we now acknowledge that these poly-synaptic circuits are likely also involved in the PAP4-induced recovery of function. To tie in with the ablation of PAP-induced EMG recovery following re-lesion combined with the relatively-low amounts of rVRG axon regeneration observed caudally past C3, it is possible that the regenerating rVRG axons that mostly reach the C3'ish spinal cord are innervating intra- and inter-segmental interneuron populations that allow these regrowing rVRG axons to also mediate inspiratory activation of more caudally-located PhMNs in the C4-C5 spinal cord via circuits that are not restricted to rVRG-PhMN monosynaptic connection.

Based on a reviewer comment below, we have also modified our circuit diagrams in Figure 1 to more accurately reflect the complexity of supraspinal input to the PhMN pool (e.g. that rVRG neurons actually provide bilateral input to the spinal cord via crossing likely at multiple levels of the neuraxis). However, while we now extensively discuss the possible role of interneurons in the effects of PAP treatment, we have chosen to not include these spinal interneurons in this diagram to focus on only the neurons and axonal pathways that we are experimentally examining in the present study.

These issues are particularly important when the quantitative data for VRG axon number and distance traveled distal to the lesion is considered (see below) - these measures both seem rather small considering the extent of physiological recovery demonstrated.

Response: Please see our response above at it directly pertains to this reviewer comment. Furthermore, we now discuss how we do not achieve robust levels of synaptic reinnervation of PhMNs, even at C3 where we observe the most robust regeneration. We discuss how this further suggests that rVRG axon regeneration may not be playing a role in functional recovery at medial and dorsal diaphragm subregions and may only be playing a partial role at the ventral subregion (or may not even be involved here as well). Nevertheless, we do still emphasize our exciting findings that, using a systemic delivery strategy, we can therapeutically achieve relatively long-distance regeneration of these critically-important rVRG axons through the lesion and back toward their PhMN targets where they synaptically reconnect (even if it is reconnection with only some PhMNs). As reviewers suggest, we no longer describe this as circuit “restoration” given that this implies a degree of reconnection that approximates the original pre-injury condition, which we have definitely not achieved.

The reviewers and reviewing editor suggest that the authors give more consideration to serotonergic and contralateral VRG sprouting as a source for distal diaphragm recovery in their discussion.

Response: As described above, we have greatly expanded our discussion of the role of serotonergic axon sprouting in functional recovery. With respect to the contribution of contralateral-originating rVRG axons, we have also expanded our discussion. While our data do not show morphological effects of PAP4 on sprouting of these fibers within the PhMN pool ipsilateral to the hemisection, they may still be playing a role in the effects of PAP4. It is not feasible to examine every possibility, so we have toned down our interpretation of the Figure 6 findings. We now state that PAP4 does not promote sprouting of these axons in the ipsilateral PhMN pool; however, it is possible that PAP4 could be, for example, altering connections between these rVRG axons and PhMNs at a synaptic level and/or that PAP4 could be modulating the proportion of synaptic connections that are functional. We have not addressed these questions; therefore, we state that future studies will be necessary to further explore the contribution of spared rVRG input to the actions of PAP4.

The differences/lack of differences across diaphragm regions are only superficially considered and should be more fully addressed. This information is important to the interpretation of axon regeneration.

Response: As described above, we now extensively discuss differences in axon plasticity across the PhMN pool and how this may impact recovery. As pointed out in this reviewer comment, we also discuss differences in EMG data across the three sub-regions. We describe how EMG amplitude in the ventral region is not significantly different than uninjured control, while in the dorsal and medial regions the recovery (which is still significantly greater than DMSO-only control in both regions) does not statistically reach uninjured control levels. It is possible that the greater levels of rVRG axon regeneration and PhMN reinnervation by these fibers that occurred at the most rostral PhMN locations (combined with the robust 5-HT sprouting) resulted in the enhanced recovery observed in the ventral subregion, while the reduced (but still significant) recovery observed at the medial and dorsal subregions was due to the fact that only 5-HT axon sprouting occurred at the most caudal portions of the PhMN pool.

Major and minor points from reviewers are synthesized below - though these seem extensive, the bulk could be addressed by text changes and re-analyses of existing data. The possibilities of tissues available for some of the additional analyses (e.g., spinal cords may have been imaged but not analyzed) and supporting data potentially excluded from the manuscript were raised in our online discussion.

Response: We have attempted to address every reviewer comment, including each comment that involves the request of additional experimental analyses.

Major points:

1. The extent of axon regeneration did not correspond through the full phrenic nucleus yet all parts of the diaphragm showed good recovery of function (though not acknowledged by the authors). Figure 5B shows fewer than 40 VRG axons enter the lesion zone, fewer than 30 exit the lesion zone, and fewer than 20 reach 500 um from the rostral end of the lesion zone. It appears that about 5 axons reach 1500 μm from the rostral end of the lesion zone, which the authors state is the distal end of C3. In the C3 cord the authors state there are 1.8 synapses observed per CTB- labeled PhMN but if there are only 5 or so axons that would mean maybe 10 synapses, which may not constitute significant synaptic reinnervation as is stated on page 18. The issue is the data is very interesting but somewhat over stated (i.e., not a large number of axons, not significant reinnervation, and not restoration of original circuitry, at least not solely due to VRG axons). The return of function was effectively lost when the original lesion site was re-lesioned. This is a crude but effective demonstration that a major source of recovered function was from axons growing through the lesion site. The authors recognize the need for more sensitive and cell specific approaches for future experiments. However, it is critical that serotonergic sprouting, both ipsilateral and contralateral, be given more consideration as a source of reinnervation/function that may or may not be abolished by a second lesion.

Response: We have extensively discussed this comment in our responses above to the reviewer comments in the “Synthesis Statement”. We now discuss how we do not achieve robust levels of synaptic reinnervation of PhMNs, even at C3 where we observe the most robust regeneration (though it is important to note that we are likely significantly underestimating the degree of synaptic input to PhMNs given that CTB labels only the soma and proximal dendritic processes of these PhMNs). We discuss how this further suggests that rVRG axon regeneration may not be playing a role in functional recovery at medial and dorsal diaphragm subregions and may only be playing a partial role at the ventral subregion (or may not even be involved here as well). Nevertheless, we do still emphasize our exciting findings that, using a systemic delivery strategy, we can therapeutically achieve relatively long-distance regeneration of these critically-important rVRG axons through the lesion and back toward their PhMN targets where they synaptically reconnect (even if it is reconnection with only some PhMNs). As reviewers suggest, we no longer describe this as circuit “restoration” given that this implies a degree of reconnection that approximates the original pre-injury condition, which we have definitely not achieved.

We now provide a more nuanced interpretation that highlights the likely contribution of serotonergic input (both 5-HT fibers injured by the initial injury and re-lesion and those spared by the two lesions) and does not overstate the contribution of rVRG axon regeneration. We discuss how serotonergic axon growth may instead be the plasticity mode operating across the PhMN pool to drive recovery at all three diaphragm sub-regions given that we observed equivalent levels of 5-HT axon synaptic innervation of PhMNs at all three caudal distances from the lesion. In addition, we state that it is possible that both rVRG and serotonergic axon plasticity is contributing to functional recovery, but that the relative contribution of each may vary significantly across the PhMN pool.

2. Measurements of eupneic diaphragm EMG amplitude were used to conclude that PAP4 treatment induced a greater extent of functional recovery 8 weeks post-injury, but deficits in other responses (e.g., following exposure to maximal chemoreceptor activation) or in ventilation were not measured. It is impressive that there is significant recovery of ventral region diaphragm activation (Figure 3E) that likely is attributed to having more axons reaching C3 than either C4 or C5. Though it is difficult to evaluate the importance of the small change primarily in one section of the diaphragm, the data show activity in the medial and dorsal diaphragm regions that is significantly better than DMSO treated animals but still considered to be no recovery compared to intact animals by the authors. Whether this is due to reduced number of VRG axons is not clear and not discussed. This warrants further discussion about distance of regeneration and assignment of functional activity based solely upon the re-lesion experiment. Also, several rat atlases indicate that the C3 segment of spinal cord is about 3.5 mm in length (same for C4 and C5), so there is some confusion here about what constitutes C3, C4 and C5 levels in this study.

Response: We apologize for not being clear. We are not saying that recovery did not occur in the medial and dorsal subregions of the diaphragm; we are only stating that recovery in these two subregions did not statistically reach uninjured control levels, while the recovery observed in the ventral subregion was not statistically different than uninjured levels. We have attempted to make this point clearer in the revised manuscript.

With respect to the distance issue, we find with our CTB labeling in fixed sagittal spinal cord sections that the CTB+ PhMN pool extends approximately 6mm in length in the rostral-caudal axis. We also measured fixed unsectioned whole cervical spinal cord and found that measuring C3 root to C5 root was in the same range (approximately 7mm in rostral-caudal length).

3. Strong concluding statements regarding the causal relationship of rVRG axon regeneration to recovery following PAP4 treatment are still present in the Abstract, New & Noteworthy, and Introduction sections. Several interpretation issues raised in the previous and current reviews regarding the underlying mechanisms for the observations in this report are present. Strongly worded concluding statements in the Results are also not justified.

Response: As we extensively discussed above in our responses to the reviewer comments in the “Synthesis Statement”, we have significantly altered the manuscript throughout the document to uniformly tone down (and in many cases dramatically alter) our interpretations and conclusions, in particular the questioned role of rVRG axon regeneration in causally driving functional recovery.

4. Changes in phospho-S6 kinase immunofluorescence 8 weeks after injury are interpreted as a direct consequence of PAP4 treatment weeks before, yet the mechanism is likely complex given that PAP4 treatment occurred weeks earlier, information regarding the pharmacology of PAP4 is limited, and treatment was systemic and not selective to one cell type (e.g., rVRG neurons vs. surrounding glia). These considerations limit the interpretation of underlying mechanism and were identified by previous reviewers. The authors have not sufficiently moderated their concluding statements throughout the manuscript and not just in the discussion (e.g., abstract, introduction, results sections suggest that the evidence provided in the manuscript is conclusive; e.g., page 5 - last paragraph; page 15 - end of 3rd paragraph).

Response: Again, we have now significantly altered the manuscript throughout the document to uniformly tone down (and in many cases dramatically alter) our interpretations and conclusions, in particular the questioned role of rVRG axon regeneration in causally driving functional recovery. As pointed out specifically in this reviewer comment, we have also moderated our interpretation of the pS6 kinase data. While we do observe increased pS6K levels in rVRG neurons with PAP4, we have not shown that this effect is directly on rVRG neurons, particularly considering the long duration between the end of peptide treatment and the time of analysis.

5. Interpretation of the lack of effects of PAP4 treatment on glial scarring or injury severity is limited to a single averaged measurement of the maximal length of the GFAP scar in 3 animals/group. This seems insufficient to support the authors' conclusion. Do they have other data to support this conclusion or they could present these data as suggestive emphasizing additional experiments are needed for conclusive evidence.

Response: As suggested by the reviewer comment, we now present these scar data as suggestive of the need for additional experiments to more conclusively rule out the possibility that PAP4 affected glial scar response.

6. Strongly worded concluding statements in the Results should be moderated in accordance with these considerations (e.g, page 17 - “PAP4 promoted robust, long-distance rVRG axon regeneration through the lesion and into caudal spinal cord” or page 20 - “Re-lesion ablated PAP4-induced restoration of diaphragm function”).

Response: We have toned down all of the concluding statements in the text of the Results section.

7. Important information for the field is not consistently reported or considered. For example, ipsilateral and contralateral- originating rVRG axonal input to the PhMNs is simply not shown. Furthermore, this type of information may help inform whether rVRG axons cross the midline above or below C2 (e.g., at the level of the motor pool). Furthermore, information for Figures 5 and 6 on the contralateral phrenic motor pool is important to the interpretation of directed vs. generalized axonal sprouting and to provide confidence in the robustness of the methods (e.g., the density of mCherry axons on the side of rVRG injection proximal to the lesion should be similar, etc.).

Response: We have addressed this comment in several ways.

First, we have modified our circuit diagrams in Figure 1 to more accurately reflect the complexity of supraspinal input to the PhMN pool (e.g. that rVRG neurons actually provide bilateral input to the spinal cord via crossing likely at multiple levels of the neuraxis). However, we have chosen to not include spinal interneurons in this diagram to focus on only the neurons and axonal pathways that we are experimentally examining in the present study.

In Figure 5 (i.e. the regeneration data), we performed mCherry-labeled rVRG axon counts at even more rostral distances from the lesion i.e. (up to 500um rostral to the rostral end of the lesion) and demonstrate that the number of ipsilateral-originating rVRG axons is the same between the two groups at these rostral locations, providing confidence in the AAV2-mCherry labeling protocol (i.e. that we have equivalent labeling of rVRG neurons in both experimental groups).

With respect to the issue of effects on axon growth within the contralateral PhMN pool, we now provide quantification of sprouting of rVRG fibers in the C3 ventral horn contralateral to the hemisection in animals injected with AAV2-mCherry into the contralateral rVRG. PAP4 treatment increased the number of mCherry-labeled axons originating in the contralateral rVRG within the contralateral C3 ventral horn, demonstrating that - while PAP4 did not have effects on these spared rVRG axons within the ipsilateral PhMN pool - systemic delivery of a PTEN inhibitor can stimulate plasticity of spared bulbospinal axon populations in the spinal cord.

Similarly, we quantified 5-HT axon sprouting in the contralateral PhMN. PAP4 treatment also stimulated serotonergic axon sprouting in the contralateral PhMN pool, demonstrating that effects of PAP4 on 5-HT axon plasticity were not restricted to only the side of injury

8. Similar considerations for serotonergic innervation of the PhMN pool to those raised in point # 7 apply. Please, consider reporting of findings in the PhMN pool contralateral to injury.

Response: As described above in our response to reviewer comment #7, we quantified 5-HT axon sprouting in the contralateral PhMN.

9. The discussion section should reflect the limitations resulting from the methodological issues identified in this (and the previous) review. Concluding statements regarding the degree of functional loss (and restoration) in the C2 hemisection model should be revised in light of the available evidence regarding functional impairment. This point is particularly relevant to the present study given the very restricted set of functional measures used.

Response: As described above in response to other reviewer comments, we revised our various concluding statements throughout the manuscript based on our new discussion of the observed neuroanatomical and functional changes.

In addition, we now acknowledge the limitations of our functional analysis. We conducted electrophysiological recordings (i.e. EMGs and CMAPs) to specifically assess effects on diaphragm activation and PhMN-diaphragm circuitry, though EMGs and CMAPs do not fully provide evaluation of the physiological effects on respiratory function. Importantly, we did not perform assessments of overall ventilatory function such as whole-body plethysmography or blood gas measurements. In addition, we did not conduct EMG recordings in response to hypercapic and hypoxic challenges as in some of the previously published studies using similar cervical SCI models. To further examine the functional effects of our PAP4 approach on respiratory circuitry, in future work we will need to test effects on these additional functional outcomes and in response to respiratory challenges.

Minor points:

1. Analyses usually involve multiple groups and measurements within an animal, yet the statistical information provided does not sufficiently identify the comparisons being performed. Mostly post hoc p values are provided when there are multiple comparisons performed even within an animal (e.g., multiple sites, side of injury, level of the spinal cord, etc).

a) Please, report all measurements and p-values with consistent precision (e.g., one decimal point for lesion length measurements and average fluorescence intensity/cell - pages 15 and 20). Three decimal points for the proportion of NMJ types is clearly not justified (page 17). Statistical significance levels are also inconsistently presented throughout the Results section.

Response: We have modified the reporting of p values throughout the manuscript.

b) Please, indicate clearly and justify when measurements are not averaged within an animal (e.g., pS6k immunofluorescence was compared across groups using the number of cells, but significant differences across animals are likely to be present).

Response: The only case in which measurements were not averaged within an animal to produce a single value for a given animal was for the pS6K quantification. We choose to perform the analysis in this manner as we observed significant neuron-to-neuron variability in pS6K levels within an animal. Obtaining a single mean value for the entire animal does not reflect the shift we see in individual neurons across the entire population within an animal. Importantly, changes in this intracellular signaling can have significant effects on functional outcome even if only a subset of these rVRG neurons have alterations in PTEN signaling and consequent effects on axonal growth capacity.

c) There is insufficient clarity regarding the analysis of diaphragm EMG activity (pg. 16; and, Fig. 3). Measurements were performed over 5 min across three sections on each side of the diaphragm for each animal, yet the F values provided do not clearly articulate what comparisons are being conducted; only post-hoc Tukey details are provided).

Response: For explaining which statistical comparisons are being conducted, we have now tried to make this clearer in the text of the Results.

We now provide in the Methods section the details of our EMG recording and subsequent analysis. For each subregion of the diaphragm, it usually takes a minute or so to get the EMG recording going well (e.g. to position the electrodes to get a clean signal with no background). Once we begin obtaining a good signal, we record for 60 seconds continuously (not for 5 minutes; we have corrected this in the manuscript), and we then move onto the next subregion. During analysis, we then we analyze every inspiratory burst over these 60 continuous seconds for each subregion. Importantly, the person conducting the EMG recordings (as well as the subsequent quantitative analysis) was always completely blinded to group identity and we randomly tested animals from different groups on the same day.

2. Please report other ventilatory parameters (e.g., respiratory rate & duty cycle both of which could be derived from the EMG). It is unfortunate that they seemingly did not perform additional functional studies (e.g., with maximal chemoreceptor stimulation). As a result, any functional improvement was incompletely studied and the present study is more promising that conclusive of its potential therapeutic role.

Response: We now provide quantification of both respiratory rate and duty cycle for the EMG data presented in Figure 3.

As stated above in response to reviewer comment #9, we now acknowledge the limitations of our functional analysis (i.e. that we only conducted electrophysiological recordings and did not perform assessments of overall ventilatory function such as whole-body plethysmography or blood gas measurements).

3. The Methods section could be shortened if measurements conducted with similar techniques were aggregated (e.g., immunohistochemical analyses). Several additional methodological issues deserve attention, some of which also detract from the confidence in the authors' interpretation of their findings.

Response: We feel that unique details relevant to specific analyses are provided in the different sections of the Methods pertaining to immunohistochemistry; therefore, we feel that these separate sections should be kept as it could make it more confusing to compile all of these methods together into a single long section.

a) Please, carefully review report of the timing of intramedullary AAV injection. The text (pg 15) and figure 1 legend are inconsistent (1 vs. 2 weeks post-injury).

Response: We have corrected this throughout. The intra-brainstem AAV injection was performed at 2 weeks post-injury.

b) Also, report for timing of CMAP measurements and intrapleural CTB injection are inconsistent (text pg 15 - week 7 post-injury vs. Figure 1 legend - 4 days before terminal = 8 weeks).

Response: We have corrected this throughout. The CMAP recordings and CTB injection were performed at 4 days pre-sacrifice.

c) Measurements of synaptic contacts on neurons are presented as contacts/labeled motor neuron, yet there are likely differences in the motor neuron surface area that was examined. Accordingly, please provide indicate whether only motor neuron soma were examined and if there were considerations regarding the extent of membrane surface area studied.

Response: Only CTB-labeled motor neuron cell bodies were examined in this quantitative analysis; membrane surface area was not considered.

d) Please, provide the orthogonal projections for Fig. 7M.

Response: We now provide the orthogonal image.

e) The representative tracings in Fig 8A-B do not appear to match the summary data for the ventral diaphragm DMSO group.

Response: We now provide new representative images for the DMSO-only condition that match the quantification by showing the greater degree of recovery in this group that we find in the ventral region even with the DMSO-only treatment.
